# The anti-circumsporozoite antibody response to repeated, seasonal booster doses of the malaria vaccine RTS,S/AS01_E_

**DOI:** 10.1038/s41541-025-01078-0

**Published:** 2025-02-06

**Authors:** M. Sanni Ali, Lisa Stockdale, Issaka Sagara, Issaka Zongo, Rakiswendé Serge Yerbanga, Almahamoudou Mahamar, Frédéric Nikièma, Amadou Tapily, Frédéric Sompougdou, Modibo Diarra, Duncan Bellamy, Samuel Provstgaard-Morys, Charles Zoungrana, Djibrilla Issiaka, Alassane Haro, Koualy Sanogo, Abdoul Aziz Sienou, Mahamadou Kaya, Seydou Traore, Oumar M. Dicko, Youssouf Kone, Hama Yalcouye, Ismaila Thera, Kalifa Diarra, Paul Snell, Opokua Ofori-Anyinam, Chris Ockenhouse, Cynthia Lee, Katie Ewer, Halidou Tinto, Abdoulaye Djimde, Jean-Bosco Ouedraogo, Alassane Dicko, Daniel Chandramohan, Brian Greenwood

**Affiliations:** 1 London School of Hygiene & Tropical Medicine, London, United Kingdom; 2 Jenner Institute, University of Oxford, Oxford, UK; 3 The Malaria Research and Training Center, University of Sciences, Techniques and Technologies, Bamako, Mali; 4 Institut de Recherche en Sciences de la Santé, Bobo-Dioulasso, Burkina Faso; 5 Institut des Sciences et Techniques, Bobo-Dioulasso, Burkina Faso; 6 GSK, Wavre, Belgium; 7 PATH, Seattle, USA; 8 GSK Vaccines Institute for Global Health, Sienna, Italy

**Keywords:** Infectious diseases, Vaccines

## Abstract

The recently deployed RTS,S/AS01_E_ malaria vaccine induces a strong antibody response to the circumsporozoite protein (CSP) on the surface of the *Plasmodium falciparum* sporozoite which is associated with protection. The anti-CSP antibody titre falls rapidly after primary vaccination, associated with a decline in efficacy, but the antibody titre and the protective response can be partially restored by a booster dose of vaccine, but this response is also transitory. In many malaria- endemic areas of Africa, children are at risk of malaria, including severe malaria, until they are five years of age or older and to sustain protection from malaria for this period by vaccination with RTS,S/AS01_E_, repeated booster doses of vaccine may be required. However, there is little information about the immune response to repeated booster doses of RTS,S/AS01_E_. In many malaria-endemic areas of Africa, the burden of malaria is largely restricted to the rainy season and, therefore, a recent trial conducted in Burkina Faso and Mali explored the impact of repeated annual booster doses of RTS,S/AS01_E_ given immediately prior to the malaria transmission season until children reached the age of five years. Anti-CSP antibody titres were measured in sera obtained from a randomly selected subset of children enrolled in this trial collected before and one month after three priming and four annual booster doses of vaccine using the GSK ELISA developed at the University of Ghent and, in a subset of these samples, by a multiplex assay developed at the University of Oxford. Three priming doses of RTS,S/AS01_E_ induced a strong anti-CSP antibody response (GMT 368.9 IU/mL). Subsequent annual, seasonal booster doses induced a strong, but lower, antibody response; the GMT after the fourth booster was 128.5 IU/mL. Children whose antibody response was in the upper and middle terciles post vaccination had a lower incidence of malaria during the following year than children in the lowest tercile. Results obtained with GSK ELISA and the Oxford Multiplex assay were strongly correlated (Pearson’s correlation coefficient, r = 0.94; 95% CI, 0.93–0.95). Although anti-CSP antibody titres declined after repeated booster doses of RTS,S/AS01_E_ a high, although declining, level of efficacy was sustained suggesting that there may have been changes in the characteristics of the anti-CSP antibody following repeated booster doses.

**Clinical Trials Registration**. NCT03143218.

## Introduction

Two malaria vaccines, RTS,S/AS01_E_ and R21/Matrix-M, have recently been approved for deployment in malaria endemic areas by the World Health Organisation (WHO)^1,2^. The recommended dosing schedule for each vaccine is three priming doses administered one month apart, followed by a booster dose administered 12 months later. WHO has recommended that a further, seasonal booster dose may be considered in areas where malaria transmission is highly seasonal as a high level of protection is provided for only a first few months after the administration of three priming doses or a subsequent booster dose of RTS,S/AS01_E_^3,4^. In many of the areas in sub-Saharan Africa with seasonal malaria transmission, the risk of malaria, including severe malaria, remains high throughout the first five years of life. Hence, repeated booster doses of RTS,S/AS01_E_ are likely to be required to achieve a high level of protection throughout this period of high risk. The effectiveness of this approach has been demonstrated in a trial conducted over a five-year period (2017–2021) in Burkina Faso and Mali in which children who had received three priming doses, were given three or four annual seasonal booster doses of RTS,S/AS01_E_ with or without Seasonal Malaria Chemoprevention SMC^5,6^. Repeated seasonal booster doses of RTS,S/AS01_E_ were non-inferior to SMC given alone, an intervention which provides 70–80% protection against uncomplicated and severe malaria when given under trial conditions^7^, and the protective efficacy of the combination as compared with chemoprevention alone was 57.7% (95% CI, 53.3 to 61.7) against clinical episodes of malaria, 66.8% (95% CI, 40.3 to 81.5) against hospital admission with severe malaria, and 66.8% (95% CI, −2.7 to 89.3) against death from malaria^6^.

Three priming doses of RTS,S/AS01_E_ vaccine induce a strong antibody response to the circumsporozoite protein (CSP) of *Plasmodium falciparum* but this wanes rapidly. A booster dose partially restores the anti-CSP antibody response but not to the level seen after the priming doses^3,4^. Thus, there has been concern that repeated booster doses of RTS,S/AS01_E_ might lead to a progressive decline in antibody response and declining efficacy. In the trial of seasonal booster vaccination with the RTS,S/AS01_E_ vaccine conducted in Burkina Faso and Mali, lower anti-CSP antibody responses were seen after first and second booster doses of RTS,S/AS01_E_ than after the three priming doses^8^. Measurement of the anti-CSP antibody response to additional booster doses of RTS,S/AS01_E_ has been undertaken to help in determination of how frequently booster doses of the RTS,S/AS01_E_ vaccine should be given, an important issue currently being considered by the WHO and other policy making organisations and this is described in this paper.

Now that two malaria vaccines RTS,S/AS01_E_ and R21/Matrix-M have been shown to provide an important degree of protection against clinical malaria^9,10^, and both have been approved by WHO^1,2^, malaria endemic countries are faced with a decision as to which vaccine to choose for their national programme. No head-to-head efficacy trial of the two vaccines has been conducted, and comparisons of the antibody response to the two vaccines are difficult to make because different anti-CSP antibody assays have been used in evaluation of the response to each vaccine. Most serological evaluations of the anti-CSP antibody response undertaken in clinical trials of the RTS,S/AS01_E_ vaccine have employed an ELISA developed at the Centre for Vaccinology at Ghent University, Belgium (CEVAC) for GSK^11^. In contrast, a novel multiplex MSD assay developed at Oxford has been used to assess the immune response in a Phase 3 R21 clinical trial^12,13^. In this paper, anti-CSP antibody responses to repeated booster doses of the RTS,S/AS01_E_ vaccine as measured using the GSK ELISA and the MSD assay are presented and compared.

## Results

### Study children

Children for inclusion in the serology study were selected at random by an independent statistician using systematic random sampling after sorting to give implicit stratification by age and gender as described in the methods section of the paper. On average, 150 children in the RTS,S/AS01_E_ alone group (range per year 99–192) and 147 (range 99–193 per year) in the RTS,S/AS01_E_ + SMC combined group were recruited to the serological sub-study each year. In each year, a small number of children in the SMC alone group (mean 30; range 19–38 between years) were recruited to determine the background titre of anti-CSP antibody resulting from natural exposure to malaria infection. Table 1 shows the number of pre- and post-vaccination paired blood samples obtained from study children by treatment arm in each year of the trial. Gender was well balanced with 50.2% of children being male and 49.8% female. Both gender and age were distributed similarly when stratified by country (Supplementary Table 1). The mean age and gender of children who were included in the sub-sample of children whose samples were tested with the Oxford MSD assay in each study year are shown by study group in Supplementary Table 2.

**Table 1:** Age in months, and gender of children at the time of collection of pre-vaccination blood samples in each year of the study, for both countries (Burkina Faso and Mali) combined

Study Children’s Characteristics	Contact	SMC Alone		RTS,S Alone		RTS,S + SMC		Both RTS,S Groups Combined	
		*N*	Mean (SD), %	*N*	Mean (SD), %	*N*	Mean (SD), %	*N*	Mean (SD), %
**Both countries**									
Age, Mean (SD)	Pre-2017	29	13.4 (4.15)	102	12.3 (4.39)	99	12.3 (4.18)	202	12.3 (4.28)
Male Sex, Percent		11	37.9	50	49	49	49	99	49
Age, Mean (SD)	Pre-2018	38	24.8 (4.53)	141	25.5 (4.24)	138	25.1 (4.19)	279	25.3 (4.21)
Male Sex, Percent		17	44.7	73	51.8	74	53.6	147	52.7
Age, Mean (SD)	Pre-2019	36	37 (4.31)	153	36.9 (3.91)	138	36.9 (3.98)	291	36.9 (3.94)
Male Sex, Percent		21	58.3	72	47.1	65	47.1	137	47.1
Age, Mean (SD)	Pre-2020	19	49.2 (3.81)	162	49.8 (4.19)	165	49.8 (4.2)	327	49.8 (4.19)
Male Sex, Percent		10	52.6	85	52.5	85	51.5	170	52
Age, Mean (SD)	Pre-2021	28	57.8 (1.78)	192	57.9 (1.68)	193	57.8 (1.68)	385	57.8 (1.68)
Male Sex, Percent		14	50	100	52.1	95	49.2	195	50.6

*N* total numbers, *SD* standard deviation, *SMC* seasonal malaria chemoprevention.

#### IgG anti-CSP antibodies titres measured by GSK ELISA following priming and booster doses and their relationship to protection against malaria infection

**Fig. 1:**
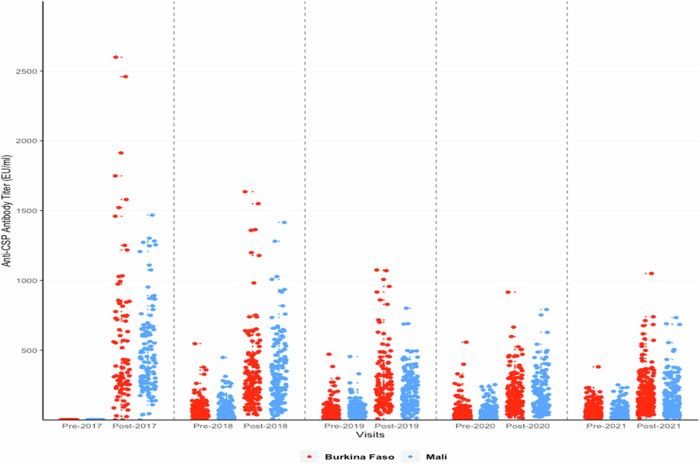
Anti-CSP antibody responses to priming and booster doses of the RTS,S/AS01_E_ malaria vaccine. Anti-CSP IgG antibody titres as measured by the GSK ELISA in individual children pre- and post-priming vaccination (2017) and pre and post first (2018), second (2019), third (2020) and fourth (2021) booster seasonal vaccination doses shown by country. Results from Burkina Faso are shown in red, those from Mali in blue. CSP circumsporozoite, EU enzyme linked immunosorbent assay unit.

**Fig. 2:**
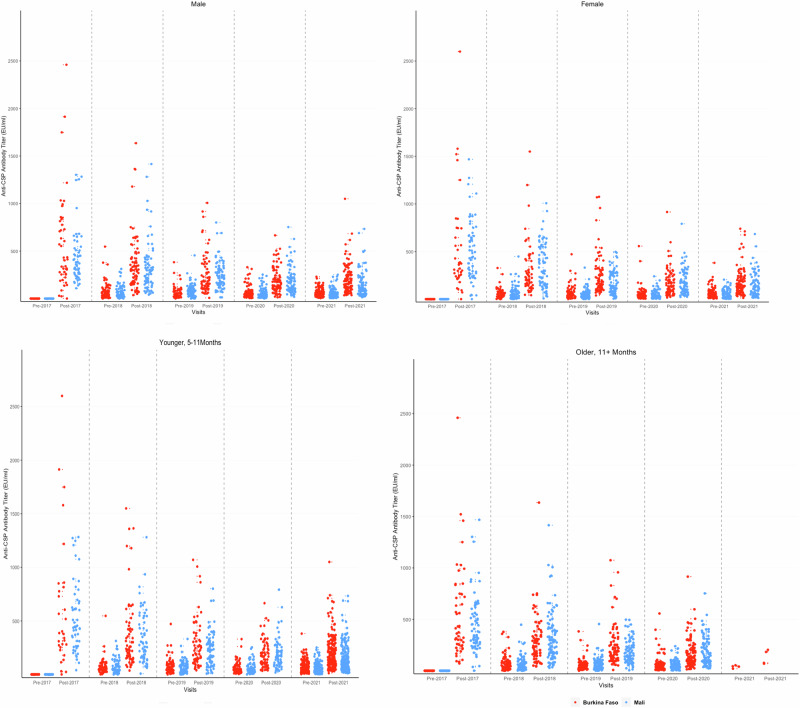
Anti-CSP antibody responses to priming and booster doses of the RTS,S/S01_E_ malaria vaccine by gender and age at the time of vaccination. Anti-CSP antibody titres measured by the GSK ELISA in individual children pre- and post-priming vaccination (2017) and pre and post first (2018), second (2019), third (2020) and fourth (2021) booster seasonal vaccination doses are shown by country. Results from children in the Burkina Faso study are shown in red, children in the Mali study are shown in blue. The top panels show titres of male and female children, and the lower panels show titres of younger and older children (age at first vaccination). CSP circumsporozoite, EU enzyme linked immunosorbent assay unit. Only a few children who had been aged 11 months of age at the time of first vaccination were eligible for vaccination in 2021, having reached the age of five years when they were no longer eligible to receive RTS,S/AS01_E_ or SMC in 2021. Consequently, there were only a few vaccinated children in the randomly selected group of children for the immunology serology sub-study in 2021 who had been 11 months old or older at the time of first vaccination.

Anti-CSP ELISA IgG antibody titres measured at the CEVAC laboratory at the University of Ghent before and after priming and booster doses of the RTS,S.AS01_E_ vaccine are shown in Table 2 and Fig. 1; results stratified by country are shown in Supplementary Table 3. Geometric Mean Titres (GMTs) obtained one month after each booster dose were lower than that obtained one month after the third priming dose and declined substantially year by year; the GMT after the third priming dose was 368.9 EU/ml (95%CI, 318–428), the GMT after the first booster dose 257.5 EU/ml (95%CI, 234.6–282.7) and the GMT after the fourth booster dose 128.5 EU/ml (95%CI, 118.4–139.6). The fold ratio increase in titre after three priming doses and four subsequent booster doses (the geometric mean ratio of post – pre vaccination titre) was 446.46 (362.07 to 550.51) after the three priming doses and 5.81 (4.92–6.86), 3.87 (3.39 to 4,42), 3.47(3,14 to 3,83) and 3.18 (2.92 to 3.46) after each subsequent booster dose. A Pearson correlation analysis of the correlation between the pre- and post-vaccination log_10_ transformed titres was undertaken. The correlation coefficients and 95% CI for the first and successive booster doses were 0.82 (0.78 to 0.86), 0.84 (0.81 to 0.87), 0.86 (0.83 to 0.89) and 0.90 (0.88 to 0.92) respectively. It was not possible to calculate the correlation coefficient for the response to the priming dose as the standard deviation was zero. There was no difference in the pattern of antibody response between children who had received RTS,S/AS01_E_ with or without SMC at any timepoint (Supplementary Fig. 1) and consequently these groups are combined in subsequent analyses. Responses to priming and booster doses were similar in Burkina Faso and Mali (Fig. 1), between male and female children, and between younger and older children at the time of recruitment (Fig. 2). Children in the SMC alone group had very low anti-CSP antibody titres at any timepoint and consequently are not included in the correlate of protection analyses. Comparison of the rise in anti-CSP antibody titre following vaccination as measured by the GSK ELISA across the years and by country is presented in Supplementary Table 4.

**Table 2:** Anti-CSP IgG antibody titres pre- and post-vaccination in each year of the study in children who received RTS,S/AS01_E_ vaccine with or without SMC as determined by the GSK ELISA for both countries (Burkina Faso and Mali) combined

Time of sample collection	*N*	Geometric Mean Titre, EU/ml (95% CI)	Number with 2-fold increase in titre (%)	Number with 10-fold increase in titre (%)
Both Countries				
Pre-2017	201	0.9		
Post-2017	198	368.9 (318–428)	194/197 (98.5)	194/197 (98.5)
Pre-2018	279	42.4 (37.1–48.5)		
Post-2018	279	257.5 (234.6–282.7)	247/279 (88.5)	76/279 (27.2)
Pre-2019	291	44.3 (39.1–50)		
Post-2019	291	177.4 (161.5–195)	246/291 (84.5)	31/291 (10.7)
Pre-2020	327	39.8 (36–44)		
Post-2020	327	137.5 (126.4–149.5)	265/327 (81)	25/327 (7.6)
Pre-2021	385	40.8 (37.4–44.6)		
Post-2021	381	128.5 (118.4–139.6)	299/381 (78.5)	16/381 (4.2)

Results are from all children vaccinated with RTS,S/AS01_E_ (i.e., RTS,S/AS01_E_ alone and combined groups pooled). At the pre-vaccination contact in 2017, one sample from Mali did not provide a conclusive result in the enzyme-linked immunosorbent assay. At the post-vaccination contact in 2017, four samples from Burkina Faso did not provide a conclusive result due to an insufficient volume of serum for the assay to be tested. There were no inconclusive results in the years 2018, 2019, and 2020. Four post-vaccination samples in 2021 gave inconsistent results when tested on two occasions and one sample could not be traced.

*CI* confidence interval, *EU* enzyme-linked immunosorbent assay unit.

The risk of clinical episodes of malaria in the year following priming and each booster dose of vaccine in relation to the anti-CSP titre at the start of the malaria transmission season is shown in Table 3 in which antibody titres are shown by tercile. Over the five years of the study, the incidence of clinical malaria was lower in children with an anti-CSP IgG antibody titre in the highest tercile compared to that in children with a titre in the lowest tercile (Incidence rate per 1000 person years at risk (PYAR), 191.6 [95%CI, 154.1–235.6] and 354 [95% CI, 301.3–413.3]) respectively. The hazard ratio of clinical malaria among children in the highest terciles versus lowest terciles of anti-CSP IgG antibody titers was 0.49 [95%CI, 0.37–0.66] (*p*-value <0.001). Severe malaria requiring hospital admission was infrequent in the sub-set of children in the serology sub-study (15 children) so that it was not possible to measure the potential impact of anti-CSP titre on the risk of severe malaria. Supplementary Table 5 shows the relationship between post-vaccination anti-CSP IgG antibody titre one month after priming vaccination and after each of the four booster vaccinations and the prevalence of asymptomatic malaria parasitaemia approximately five months later, at the end of the malaria transmission season. No protective effect was seen.

**Table 3:** Incidence of episodes of clinical malaria overall and by study year in children in the year after they received priming or booster doses of RTS,S/AS01_E_ with or without SMC in relation to the anti-CSP antibody titre measured one month after vaccination using the GSK ELISA

Anti-CSP antibody titre by tercile	PYAR^a^	Events	Rate per 1000 PYAR (95% CI)	Hazard Ratio (95% CI)	*P*-value
**Overall**					
Lowest	454.1	160	352.4 (299.9–411.4)	Reference	
Medium	463.2	124	267.7 (222.6–319.2)	0.68 (0.51–0.91)	0.009
Highest	472.6	90	190.4 (153.1–234.1)	0.49 (0.37–0.66)	<0.001
**Post-priming vaccination**					
Lowest	53.9	12	222.6 (115–388.9)	Reference	
Medium	58.5	3	51.3 (10.6–149.9)	0.19 (0.06–0.67)	0.009
Highest	56.4	1	17.7 (0.4–98.7)	0.06 (0.01–0.45)	0.007
**Post-first booster dose**					
Lowest	91.3	27	295.7 (194.8–430.2)	Reference	
Medium	87.3	26	297.8 (194.6–436.4)	0.96 (0.5–1.86)	0.911
Highest	91.3	18	197.1 (116.8–311.4)	0.66 (0.33–1.29)	0.225
**Post-second booster dose**					
Lowest	89.6	35	390.6 (272.1–543.3)	Reference	
Medium	93.3	20	214.3 (130.9–331)	0.49 (0.25–0.96)	0.038
Highest	96.9	11	113.5 (56.7–203.1)	0.26 (0.12–0.55)	0.001
**Post-third booster dose**					
Lowest	106.7	30	281.1 (189.6–401.3)	Reference	
Medium	103.3	34	329.1 (227.9–459.9)	1.16 (0.62–2.14)	0.646
Highest	107	29	271.1 (181.6–389.4)	0.96 (0.52–1.79)	0.909
**Post-fourth booster dose**					
Lowest	112.5	56	497.7 (375.9–646.3)	Reference	
Medium	120.8	41	339.3 (243.5–460.3)	0.49 (0.3–0.81)	0.006
Highest	120.9	31	256.4 (174.2–363.9)	0.42 (0.25–0.71)	0.001

^a^*PYAR* person years at risk. Cox regression models for the pooled analysis over all 5 years of the study were adjusted for study year, SMC, and the age and sex of the child. The overall analysis aggregates person-time and events for the terciles defined separately in each year and children above and below the specific threshold in each year of the study.

Reverse cumulative plots of antibody responses to primary and booster doses of vaccine are shown in Supplementary Fig. 2. Comparison of the incidence of clinical malaria between children with titres (as measured by GSK ELISA) above and below a putative threshold protective titre, estimated from the reverse cumulative plots as described in the methods section, were 266.8 EU/ml in 2017, 207.2 EU/ml in 2018, 157.8 EU/ml in 2019, 148.2 EU/ml in 2020 and 139.8 EU/ml in 2021 (Supplementary Table 6). Over the five years of the trial, the risk of clinical episodes of malaria was substantially less in children whose antibody titre was above the threshold than in children in whom it was below this threshold (HR 0.65 [95% C1 0,51, 0.83]).

#### Antibody responses in sera measured by the ELISA-based and by the Oxford MSD assay

GMTs measured by the Oxford MSD assay one month after each booster dose were lower than those obtained one month after the third priming dose and declined substantially year on year, as seen when antibody titres were measured with the GSK ELISA (Table 4). The impact of boosting on antibody response fell over time, in both Burkina Faso and Mali (Supplementary Table 7) in a similar way to that seen with the GSK ELISA (Table 4 and Supplementary Table 8).

**Table 4:** Logarithm of anti-CSP IgG antibody titres pre- and post-vaccination in each year in children who received RTS,S/AS01_E_ vaccine for both countries (Burkina Faso and Mali), measured using the two assays: - GSK ELISA and Oxford MSD assay

Time of Sample Collection	*N*	Log GMT, EU/ml (95% CI)	Number with 2-fold Increase in titre (%)	Number with 10-fold increase in titre (%)
**GSK ELISA (Total** ***N*** = **1484)**				
Pre-2017^a^	201			
Post-2017	198	5.9 (5.8–6.1)	194/197 (98.5)	194/197 (98.5)
Pre-2018	279	3.6 (3.5–3.8)		
Post-2018	279	5.5 (5.4–5.6)	247/279 (88.5)	76/279 (27.2)
Pre-2019	291	3.8 (3.7–3.9)		
Post-2019	291	5.1 (5–5.2)	246/291 (84.5)	31/291 (10.7)
Pre-2020	327	3.6 (3.5–3.7)		
Post-2020	327	4.9 (4.8–4.9)	265/327 (81)	25/327 (7.6)
Pre-2021	385	3.6 (3.5–3.7)		
Post-2021	381	4.8 (4.7–4.9)	299/381 (78.5)	16/381 (4.2)
**OXFORD MSD ASSAY (Total** ***N*** = **502)**				
Pre-2017	104	5.2 (5.1–5.3)		
Post-2017	114	12.1 (11.8–12.3)	109/114 (95.6)	101/114 (88.6)
Pre-2018	99	9.1 (8.8–9.5)		
Post-2018	100	11.8 (11.5–12)	97/100 (97)	51/100 (51)
Pre-2019	100	9.8 (9.6–10.0)		
Post-2019	100	11.2 (11–11.4)	77/100 (77)	16/100 (16)
Pre-2020	99	9.3 (9.10–9.50)		
Post-2020	99	10.3 (10.1–10.5)	64/99 (64.6)	4/99 (4)
Pre-2021	100	9.7 (9.4–10.0)		
Post-2021	100	10.4 (10.2–10.6)	50/100 (50)	4/100 (4)

^a^The lower limit of quantification for GSK ELISA is 1.9 EU/ml and samples with a titre below this lower limit (i.e., titre with a value of 0) were assigned a titre of 0.95 EU/ml, half the lower limit of detection. The log(0.95) = −0.051, many children have this value pre priming (in 2017) and it was not possible to calculate log GMT for these negative values.

#### Correlation between the results obtained with the GSK ELISA and the OXFORD MSD assay

**Fig. 3:**
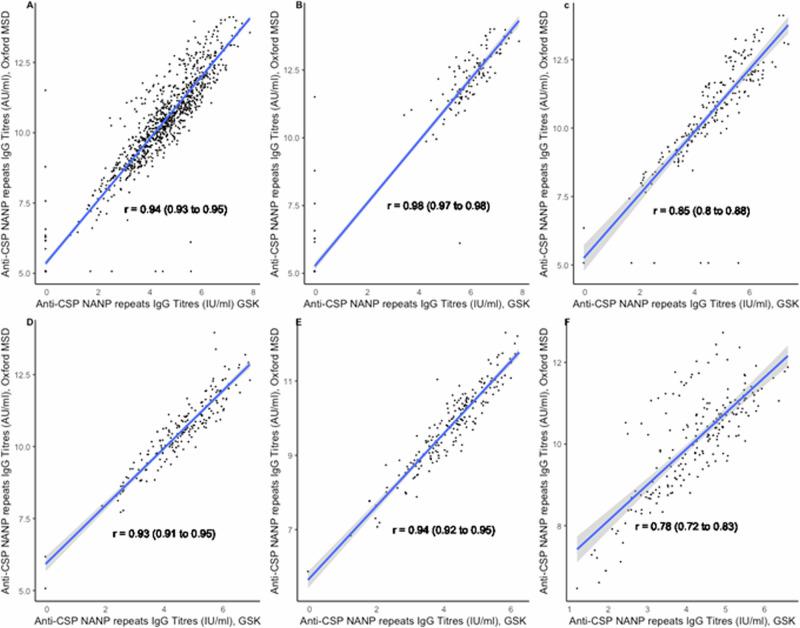
Comparison of anti-CSP antibody titres obtained in sera obtained from children vaccinated with the RTS,S/AS01_E_ vaccine using different ELISA assays. Scatterplots showing correlations between titres of anti-CSP IgG antibodies in children vaccinated with RTS,S/AS01_E_ in log10 IU/AU/mL between the GSK ELISA and Oxford MSD ELISA, combined and stratified by study year (**A**: Combined 2017–2021; **B**: 2017; **C**: 2018; **D**: 2019; **E**: 2020, and **F**: 2021).

**Fig. 4:**
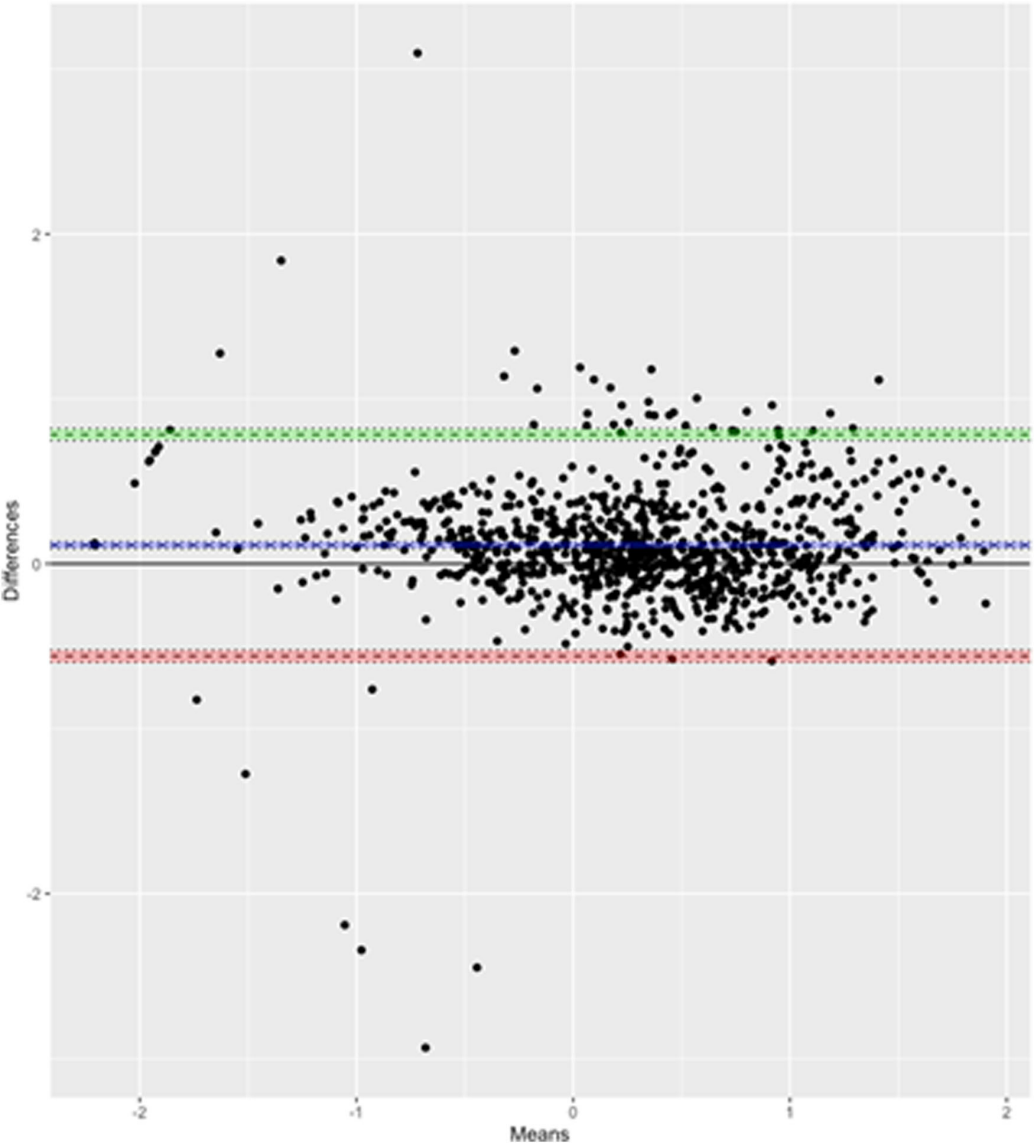
Bland-Altman plots for Oxford MSD and GSK assays. The blue line corresponds to the mean difference while the dotted horizontal green and red lines correspond to the 95% limits of agreement. The y-axis represents the difference between titres using Oxford MSD and the GSK ELISA, the X-axis represents the mean of the titres using the two assays.

Correlations between the GSK ELISA and the Oxford MSD assay were strong and significant at all timepoints (Fig. 3). The Pearson’s correlation coefficient between the two assays for all timepoints combined was 0.94 (95% CI, 0.93–0.95). Correlations varied from a low of r = 0.78 (95% CI, 0.72–0.83) in 2021 for the response to the fourth booster dose to a high of r = 0.98 (95% CI; 0.97–0.98) in 2017 following priming vaccination. Supplementary Table 9 presents the correlations between the two ELISA assays pre- and post- priming and pre- and post-booster vaccination by study year and country. Bland-Altman plots on log-transformed data show that the assays are generally in good agreement with most values within the agreement intervals. The mean differences were close to zero (−0.11 (95%CI, −0.13 to −0.09), with limits of agreement ranging from −0.78 to 0.56 (Fig. 4). There were very few outliers. The two assays showed more agreement on the post-priming or booster titres than on the pre-priming or booster titres, with limits of agreement ranging from −0.66 to 0.54 and −0.96 to 0.51 respectively.

## Discussion

Children living in the areas of sub-Saharan Africa where malaria transmission is highly seasonal need protection against malaria until they are at least five years old and, ideally, until they are even older. SMC, now widely deployed in these areas, provides substantial protection in the region of 70–80% when given under trial conditions but SMC is demanding to deliver. Vaccination provides an alternative, or complementary approach, but because of the relatively short period of high efficacy provided by the four-dose regimen of RTS,S/AS01_E_^3,4^ subsequent booster doses will be required to provide children with a high degree of protection throughout their first decade. A high level of efficacy in the first 12 months after a first booster dose of the R21-MatrixM vaccine has been observed in a phase2 trial conducted in Burkina Faso^13^ and a recent modelling paper suggests that booster doses of R21-Matrix M may provide a longer period of protection than booster doses of RTS,S/AS01_E_^14^ but it is not yet known if this protection will persist until children reach the age of five years or older or whether further booster doses may be needed with this vaccine to achieve a high, sustained level of protection.

The recent study conducted in Burkina Faso and Mali has shown that a high level of protection against clinical episodes of malaria, similar to that provided by SMC, can be achieved by annual seasonal booster doses of vaccination with the RTS,S/AS01_E_ vaccine up to the age of five years^5,6^. The sustained protection achieved over a five-year period was accompanied by increases in anti-CSP antibody titre following each annual booster dose, although the increase in titre post vaccination was substantially less than that seen after the three priming or first booster dose and showed a trend towards a lower response after each repeated booster dose. Despite declining post booster titres, efficacy against clinical malaria was sustained throughout the five-year period of the trial^6^. These findings suggest that the quality of the anti-CSP antibody may have been altered following the repeated booster doses. This may have been achieved through changes in antibody avidity and/or isotype, induction of protective antibodies binding the C terminal of the CSP molecule, antibodies binding to Fc gamma receptor promoting phagocytosis and activation of natural killer cells, or to an increase in antibodies to blood stage antigens as described in previous studies^15–23^. Some of these possibilities are currently being investigated in samples obtained during the RTS,S/AS01_E_ + SMC trial. The RTS,S/AS01_E_ vaccine also induces strong cell mediated immune response^24–29^, and interactions between antibody and cell mediated immune response have been reported^30–34^; repeated boosting with RTS,S/AS01_E_ might enhance these interactive protective responses. Unfortunately, it was not possible to investigate this possibility in the recently completed RTS,S/SAS01_E_ plus SMC trial but this should be explored. Because of the complexity of the immune response to malaria it is likely that no one response to the vaccine will provide a strong correlation of protection and that a combination of responses may be more powerful.

Why the antibody response to successive booster doses of RTS,S/AS01_E_ declines is not known. The characteristics of the antibody response to a booster dose of vaccine may be influenced by a range of variables, including the age of the recipient, the construct of the vaccine, the antigen concentration of booster vaccine, the gap between priming and booster and many other variables. Why booster doses of the RTS,S/AS01_E_ induce a lower rather than enhanced antibody response than the priming doses is not fully understood. Administration of three doses of the RTS,S/AS01_E_ vaccine with a powerful adjuvant provides a high level of stimulation to the immune and a very high concentration of anti-CSP antibody. This may have a suppressive effect on the subsequent response to re-exposure to the same antigen, but this has not been established. Establishing the mechanism for the progressive decline in antibody response to booster doses of the RTS,S/AS01_E_ vaccine, and possible ways in which this might be overcome, could enhance the potential of this and related vaccines to provide a longer period of protection.

Comparing serological results of trials of the RTS,S/AS01_E_ and R21/Matrix-M vaccines has been challenging because of differences in the assays used to assess the immune response to each vaccine as well as differences in the epidemiological situations in which the trials were conducted. This study has shown that the Oxford MSD assay used to assess the anti-NANP antibody response to the R21/Matrix-M vaccine in a phase 3 trial, shows a very similar pattern in the anti-CSP antibody response to priming and boosting with RTS,S/AS01_E_ to that observed with the GSK ELISA and a high degree of correlation was found between the two assays, as noted recently in another study^35^. Demonstration that this is the case will facilitate serological comparison between previous and future studies of the RTS,S/AS01_E_ and the R21/Matrix-M vaccines. In addition, the multiplex approach allows simultaneous measurements of antibody response against the individual components of the RTS,S/AS01_E_ and R21 antigens (central repeat region, C-terminus and HBsAg), facilitating a broader evaluation of the humoral immune response to vaccination from a small sample of serum.

As the R21/Matrix-M and RTS,S/AS01_E_ malaria vaccines become increasingly available, their use may be expanded beyond young children to other at-risk populations and in elimination campaigns. Standardisation of the methods of measurement of the antibody response to the vaccines in these different situations will be important in interpretation of the findings across these studies.

## Methods

Details of the trial during which the blood samples for the serological assays described in this paper were collected have been described previously^5,6^ and serological results obtained during the first three years of the study have also been reported^8^.

### Overall trial design, study sites and study populations

The RTS,S/AS01_E_ + SMC trial was designed to determine whether seasonal vaccination with the RTS,S/AS01_E_ malaria vaccine was non-inferior to SMC in preventing clinical episodes of malaria and/or whether the combination was superior to either intervention given alone. The primary trial endpoint was the incidence of uncomplicated clinical malaria, defined as an episode of fever (temperature ≥ 37.5 °C or a history of fever within the past 48 h), and microscopically confirmed *P. falciparum* parasitaemia at a density of 5000 parasites per µl or more in a child who presented to a health centre for treatment^5,6^ .

The trial was conducted in Bougouni and Ouélessébougou districts, Mali and in Houndé district, Burkina Faso. All households within the study areas with children aged 5–17 months on April 1^st^, 2017, were enumerated in February-March 2017. Eligible children whose parent or guardian provided written, informed consent for their child to join the trial were allocated randomly to an SMC alone, RTS,S/AS01_E_ alone, or to a combined SMC + RTS,S/AS01_E_ group by an independent statistician using permuted blocks after assorting according to age, sex, area of residence and previous receipt of chemoprevention^5,6^.

**Fig. 5:**
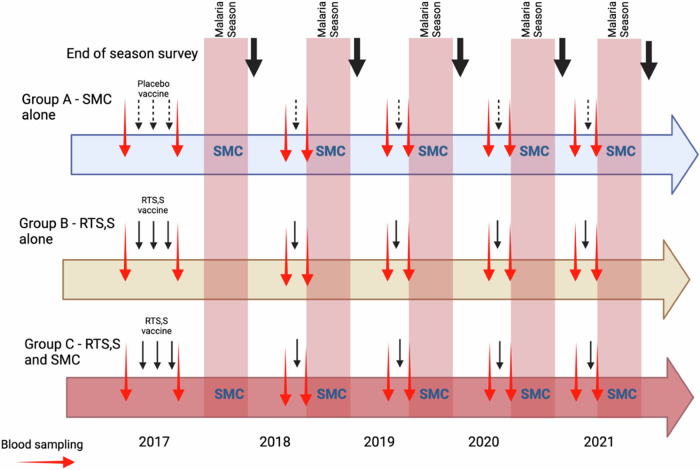
Schematic showing the interventions given to children in each of the three trial groups and their timing in relation to the malaria transmission seasons in 2017–2021. Red arrows indicate blood sampling for serology, black arrows indicate vaccination (placebo vaccines, dotted arrows; RTS,S/AS01_E_, solid arrows), and bold black arrows end of transmission season surveys.

Children in the RTS,S/AS01_E_ alone or RTS,S/AS01_E_ + SMC group received three doses of RTS,S/AS01_E_ vaccine (GSK, Rixensart, Belgium) at monthly intervals in April - June 2017 followed by seasonal booster doses in June 2018, 2019, 2020 and 2021, prior to the malaria transmission season (Fig. 5). Children in the SMC alone group received three doses of rabies vaccine (*Rabipur*^*R*^) (Bavarian Nordic A/S, Denmark) in 2017, a single dose of hepatitis A vaccine (HAVRIX^R)^) (GSK, Rixensart, Belgium) in 2018 and 2019 and booster doses of tetanus or tetanus-diphtheria toxoid vaccine in 2020 and 2021. The RTS,S/AS01_E_ + SMC and the SMC alone groups received four cycles of SMC with sulphadoxine pyrimethamine and amodiaquine (SPAQ) at monthly intervals each year until they reached the age of five years; children in Burkina Faso received an additional round of SMC in the last year of the study in line with national guidelines. Children in the RTS,S/AS01_E_ alone group received matching SMC placebo (Supplementary Table 10). All study children were given a piperonyl butoxide long lasting insecticide treated bednet at enrolment in 2017 and again in 2020.

Project staff based in study health facilities identified and treated all cases of malaria who presented at these facilities, using a Rapid Diagnostic Test (RDT), and obtained a blood film for subsequent microscopy. All admissions of study children to hospital were documented by trial staff. A cross-sectional survey for malaria prevalence was undertaken in all study children one month after the last round of SMC administration each year. Blood films were read by two independent microscopists and, in instances of a discrepancy in positivity or density, by a third reader with discrepancies resolved as described previously^36^.

#### Serology

Children for inclusion in the serology study were selected at random by an independent statistician using systematic random sampling after sorting to give implicit stratification by age and gender. The same children were sampled pre- and post-vaccination within a study year, but different children were selected each year to avoid repeat venous blood sampling of the same child over successive years.

### GSK ELISA standardised ELISA

Anti-CSP IgG antibody titres were measured by a standardised ELISA at the CEVAC laboratory Ghent University, Belgium^11^. This assay measures IgG responses to the R32LR protein which comprises two NVDP oligopeptides and thirty NANP repeats linked to the dipeptide Leu-Arg (NVDP[NANP]152LR). The lower limit of quantification for this assay is 1.9 EU/ml and samples with a titre below this lower limit (i.e., titre with a value of 0) were assigned a titre of 0.95 EU/ml, half the lower limit of detection.

### NANP multiplexed ASSAY (Oxford MSD assay)

A multiplexed assay (Oxford MSD assay) was developed by Meso Scale Discovery (MSD) and validated at the Jenner Institute, Oxford (manuscript under review). The assay simultaneously measures antibody responses to six repeats of the four-amino acid central repeat region of CSP (NANP6) in addition to the C-terminus of CSP, the full-length R21 vaccine antigen including NANP6, the C-terminus and Hepatitis B surface antigen (HBsAg), which is present in both RTS,S and R21^12^. The lower limit of quantification for this assay was 319 AU/ml and samples with a titre below this lower limit (i.e. titre with a value of 0) assigned a titre of 159.5 AU/ml, half the lower limit of detection. Staff undertaking the serological assays were blinded to the study group from which a sample came.

### Sample size and statistical analyses

The RTS,S/AS01_E_ + SMC trial recruited 6863 children (approximately 2000 in each group). Based on findings in previous RTS,S/AS01_E_ trials, it was estimated that inclusion of approximately 150 children in each country at each time point would give a study with 80% power to detect a difference of 25% in GMT between two comparison groups. For comparison of the GSK ELISA with NANP6 IgG measured by the Oxford MSD ELISA, a random sample of approximately 50 serum samples was selected from within each of the groups of children who had received the RTS,S/AS01_E_ vaccine and whose samples had been tested with the GSK ELISA.

Descriptive statistics means (standard deviations) and proportions were used to summarise demographic characteristics of children in each treatment group by year. Pre-vaccination and post-vaccination titres in each year of the study among children who received RTS,S/AS01_E_ were calculated, and geometric means and 95% confidence intervals determined. Ratios of geometric means and 95% confidence intervals were used to compare pre- and post-vaccination titres and the rise in titre after each booster dose, adjusting for SMC treatment in the combined RTS,S/AS01_E_ + SMC group.

Cox regression models were used to compare the incidence of morbidity outcomes in relation to antibody response, measured using the GSK ELISA assay, defined by terciles of the anti-CSP titres using a robust standard error to account for multiple episodes per child^37^. Reverse cumulative plots were used to define a potential cut-off titre associated with protection against episodes of clinical malaria^38^. The threshold titre was defined as the minimum titre measured in X% of children who received RTS,S/AS01_E_, where X% was the efficacy of RTS,S/AS01_E_ against clinical malaria in that year of the study. Based on these thresholds, incidence rates of clinical malaria in children with antibody titres below and above the threshold were compared in each of the study years. Poisson regression models using robust standard errors^39^ were used to estimate and compare the prevalence of *P. falciparum* infection at the end of the malaria transmission season in relation to antibody response defined by terciles of the anti-CSP titres, measured using the GSK ELISA. Pearson’s correlation coefficients and 95% Confidence intervals were used to compare the two ELISA assays. To assess agreement between assays, the limits of agreement method of Bland and Altman was used^40^. As the units were arbitrary, each assay was first standardised by subtracting its mean and dividing by the standard deviation before applying the Bland and Altman method. There was no prespecified criteria for acceptable limit of agreement for the two assays.

### Ethics and trial oversight

The trial protocol was approved by the ethics committees of the London School of Hygiene & Tropical Medicine, the Ministry of Health, Burkina Faso, the University of Sciences, Techniques and Technologies of Bamako, Mali and the trial approved by the national regulatory authorities of Burkina Faso and Mali. The trial’s Data Safety and Monitoring Board (DSMB) reviewed serious adverse events, approved the statistical analysis plan and archived the locked databases prior to unblinding. A steering committee gave scientific advice and monitored progress of the trial. Written, informed consent was obtained from the parents or guardians of all children in the trial at the start of the trial and at the start of the two-year extension period. The trial is registered on clinicaltrials.gov (https://classic.clinicaltrials.gov/ct2/show/NCT03143218).

## Supplementary information

Supplementary Tables And Figures

## Data Availability

Individual de-identified data on trial participants that were used to produce this paper, the data dictionary, protocol and the statistical analysis plan will be made available to qualified investigators following a request for use of these data which will be held at the London School of Hygiene and Tropical Medicine (https://datacompass.lshtm.ac.uk/). Data will be available from twelve months following the date of publication. Requests for access to trial data and request should be addressed to the corresponding author (brian.greenwood@lshtm.ac.uk).
