# Supplementary material for: The anti-circumsporozoite antibody response to repeated, seasonal booster doses of the malaria vaccine RTS,S/AS01E
Source: NPJ Vaccines. 2025 Feb 6;10:26. doi: 10.1038/s41541-025-01078-0 (PMC11802723; doi:10.1038/s41541-025-01078-0)
Supplement: Supplementary file 1 — Supplementary Tables And Figures [file 41541_2025_1078_MOESM1_ESM.pdf]

SUPPLEMENTARY TABLES AND FIGURES

Supplementary Table 1: Age in months, and gender of the children included in the serology study stratified by country prior to priming or booster doses of RTS,S/AS01$_E$ in each year of the study, whose samples were analysed using the GSK ELISA.

Study children's characteristics	Year	SMC alone		RTS,S alone		RTS,S + SMC		Both RTS,S groups combined	
		N	Mean (SD), %	N	Mean (SD), %	N	Mean (SD), %	N	Mean (SD), %
**Burkina Faso**									
Age, Mean (SD)	2017	17	14.2 (3.92)	43	12.7 (4.75)	43	12.6 (4.2)	87	12.6 (4.5)
Male Sex, Percent		7	41.2	23	53.5	23	52.3	46	52.9
Age, Mean (SD)	2018	16	25.6 (4.86)	65	25.1 (4.32)	67	24.8 (4.27)	132	24.9 (4.3)
Male Sex, Percent		8	50	35	53.8	38	56.7	73	55.3
Age, Mean (SD)	2019	17	36.8 (4.87)	71	37.2 (4.13)	64	37 (4.32)	135	37.1 (4.2)
Male Sex, Percent		9	52.9	34	47.9	27	42.2	61	45.2
Age, Mean (SD)	2020	10	49.2 (3.84)	79	49.8 (4.47)	90	50.1 (4.35)	169	50 (4.4)
Male Sex, Percent		6	60	43	54.4	44	48.9	87	51.5
Age, Mean (SD)	2021	17	57.6 (1.66)	103	57.8 (1.62)	108	57.6 (1.6)	211	57.7 (1.6)
Male Sex, Percent		10	58.8	52	50.5	56	51.9	108	51.2
**Mali**									
Age, Mean (SD)	2017	12	12.3 (4.38)	59	11.9 (4.11)	56	12 (4.2)	115	12 (4.1)
Male Sex, Percent		4	33.3	27	45.8	26	46.4	53	46.1
Age, Mean (SD)	2018	22	24.2 (4.29)	76	25.9 (4.16)	71	25.3 (4.12)	147	25.6 (4.1)
Male Sex, Percent		9	40.9	38	50	36	50.7	74	50.3
Age, Mean (SD)	2019	19	37.2 (3.86)	82	36.6 (3.71)	74	36.8 (3.7)	156	36.7 (3.7)
Male Sex, Percent		12	63.2	38	46.3	38	51.4	76	48.7
Age, Mean (SD)	2020	9	49.2 (4.01)	83	49.7 (3.94)	75	49.4 (4)	158	49.5 (4)
Male Sex, Percent		4	44.4	42	50.6	41	54.7	83	52.5
Age, Mean (SD)	2021	11	58.1 (2)	89	58 (1.75)	85	58.1 (1.75)	174	58 (1.7)
Male Sex, Percent		4	36.4	48	53.9	39	45.9	87	50

Abbreviations: N, total numbers; SD, standard deviation; SMC, seasonal malaria chemoprevention.

Supplementary Table 2: Age in months, and gender of the children included in the serology study in both countries (Burkina Faso and Mali) combined prior to priming or booster doses of RTS,S/AS01$_E$ in each year of the study whose samples were analysed using the Oxford MSD assay.

**Study children's characteristics**	**Contact**	**RTS,S alone**		**RTS,S + SMC**		**Both RTS,S groups combined**	
		N	Mean (SD), %	N	Mean (SD), %	N	Mean (SD), %
**Both countries**		-	-	-	-	-	-
Age, Mean (SD)	2017	56	12.5 (4.46)	48	12.4 (4.08)	104	12.5 (4.27)
Male Sex, Percent		28	50	21	43.8	49	47.1
Age, Mean (SD)	2018	49	25 (4.22)	50	24.8 (4.08)	99	24.9 (4.13)
Male Sex, Percent		24	49	30	60	54	54.5
Age, Mean (SD)	2019	50	37 (4.13)	50	36.5 (3.96)	100	36.7 (4.03)
Male Sex, Percent		23	46	20	40	43	43
Age, Mean (SD)	2020	49	49.9 (4.34)	50	49.5 (4.09)	99	49.7 (4.2)
Male Sex, Percent		26	53.1	20	40	46	46.5
Age, Mean (SD)	2021	50	57.9 (1.69)	50	57.9 (1.76)	100	57.9 (1.72)
Male Sex, Percent		24	48	27	54	51	51

Abbreviations: N, total numbers; SD, standard deviation; SMC, seasonal malaria chemoprevention.

Supplementary Table 3: Anti-CSP IgG antibody titres pre- and post-vaccination in each year in children who received RTS,S/AS01$_E$ vaccine, stratified by country and assayed using the GSK ELISA.

Time of ample collection	N	Geometric Mean Titre, EU/ml (95% CI)	Number with 2-fold increase in titer (%)	Number with 10-fold Increase in titer (%)
**Burkina Faso**				
Pre-2017	87	0.9		
Post-2017	83	349.7 (265.2-461.1)	81/83 (97.6)	81/83 (97.6)
Pre-2018	132	46.5		
Post-2018	132	256.3 (224.8-292.2)	111/132 (84.1)	31/132 (23.5)
Pre-2019	135	43.6		
Post-2019	135	200.1 (175.8-227.8)	119/135 (88.1)	21/135 (15.6)
Pre-2020	169	41.7		
Post-2020	169	137.3 (122-154.6)	135/169 (79.9)	8/169 (4.7)
Pre-2021	211	46.5		
Post-2021	209	150 (135.8-165.8)	163/209 (78)	11/209 (5.3)
**Mali**				
Pre-2017	114	0.9		
Post-2017	115	383.5 (326.6-450.3)	113/114 (99.1)	113/114 (99.1)
Pre-2018	147	39		
Post-2018	147	258.6 (226.6-295.2)	136/147 (92.5)	45/147 (30.6)
Pre-2019	156	44.8		
Post-2019	156	159.9 (139.9-182.8)	127/156 (81.4)	10/156 (6.4)
Pre-2020	158	37.9		
Post-2020	158	137.6 (122.1-155)	130/158 (82.3)	17/158 (10.8)
Pre-2021	174	34.9		
Post-2021	172	106.5 (93.5-121.4)	136/172 (79.1)	5/172 (2.9)

Supplementary Table 4: Comparison of the rise in vaccination titres post booster vaccination doses in children in the two groups who received RTS,S/AS01$_E$ (RTS,S/AS01$_E$ alone and RTS,S/AS01$_E$ +SMC combined) and by country as assessed using the GSK ELISA.

	N Pre-booster titers	Geometric Mean pre-booster titer EU/ml (95% CI)	Number post-booster titers	Geometric Mean post-booster titer EU/ml (95% CI)	Geometric Mean rise in post vs pre-booster titers (95% CI)	Ratio of Geometric Mean rise in titers (95% CI)	P value for post- vs pre-vaccination ratio
**Both Countries**							
Post-2018	279	42.4 (37.1-48.5)	279	257.5 (234.6-282.7)	6.1 (5.4-6.8)	Reference	
Post-2019	291	44.3 (39.1-50.0)	291	177.4 (161.5-195.0)	4.0 (3.6-4.4)	0.66 (0.58-0.75)	<0.001
Post-2020	327	39.8 (36.0-44.0)	327	137.5 (126.4-149.5)	3.5 (3.2-3.7)	0.57 (0.50-0.64)	<0.001
Post-2021	385	40.8 (37.4-44.6)	381	128.5 (118.4-139.6)	3.2 (3.0-3.4)	0.52 (0.46-0.59)	<0.001
**Burkina Faso**							
Post-2018	132	46.5 (38.7-55.9)	132	256.3 (224.8-292.2)	5.5 (4.6-6.6)	Reference	
Post-2019	135	43.6 (36.9-51.5)	135	200.1 (175.8-227.8)	4.6 (4.0-5.2)	0.83 (0.70-1.00)	0.047
Post-2020	169	41.7 (36.5-47.6)	169	137.3 (122-154.6)	3.3 (3.0-3.6)	0.60 (0.50-0.71)	<0.001
Post-2021	211	46.5 (41.7-51.7)	209	150 (135.8-165.8)	3.2 (3.0-3.5)	0.59 (0.50-0.69)	<0.001
**Mali**							
Post-2018	147	39.0 (32.2-47.3)	147	258.6 (226.6-295.2)	6.6 (5.7-7.7)	Reference	
Post-2019	156	44.8 (37.5-53.6)	156	159.9 (139.9-182.8)	3.6 (3.1-4.1)	0.54 (0.45-0.64)	<0.001
Post-2020	158	37.9 (32.5-44.1)	158	137.6 (122.1-155.0)	3.6 (3.3-4.0)	0.55 (0.46-0.65)	<0.001
Post-2021	174	34.9 (30.2-40.3)	172	106.5 (93.5-121.4)	3.1 (2.8-3.4)	0.47 (0.39-0.55)	<0.001

In 2018, children receive their first booster dose approximately one year after priming, in 2019 their second booster dose one year later, in 2020 their third booster dose one year later, and in 2021 their fourth booster one year later.

Supplementary Table 5: Prevalence of *Plasmodium falciparum* Infection at the end of malaria transmission season surveys, which were conducted approximately one month after the last administration of SMC and approximately five months after the last dose of priming or booster dose of vaccine was given, stratified by terciles of anti-CSP IgG antibody titres using the GSK ELISA, both countries (Burkina Faso and Mali) combined, by study year.

Anti-CSP antibody titer by tercile	Number positive for *P. falciparum*/ number tested	Prevalence % (95% CI)	Prevalence ratio (95% CI) Highest or intermediary tercile vs lowest tercile	P value for prevalence ratio
**Overall**				
Lowest	42/464	9.05 (6.44, 11.66)	Reference	
Intermediary	38/471	8.07 (5.61, 10.62)	0.79 (0.50-1.23)	0.29
Highest	27/453	6.00 (3.78, 8.14)	0.63 (0.38-1.02)	0.06
**2017**				
Lowest	1/57	1.71 (0.24, 11.4)	Reference	
Intermediary	0/62	0	0	
Highest	2/54	3.70 (0.92, 13.8)	1.61 (0.3, 22.1)	0.70
**2018**				
Lowest	10/91	11.0 (6.00, 19.3)	Reference	
Intermediary	11/90	12.2 (6.88, 20.8)	1.09 (0.46-2.62)	0.84
Highest	6/80	9.20 (3.40 15.8)	0.85 (0.29-2.29)	0.75
**2019**				
Lowest	11/88	12.5(7.04, 21.2)	Reference	
Intermediary	6/92	6.52 (2.95, 13.8)	0.43 (0.15-1.13)	0.10
Highest	5/90	5.56 (2.32, 12.7)	0.46 (0.14-1.30)	0.16
**2020**				
Lowest	11/107	10.28 (4.44, 17.03)	Reference	
Intermediary	9/106	8.49 (3.85, 15.97)	0.78 (0.31-1.88)	0.57
Highest	4/105	3.80 (0.11, 7.47)	0.35 (0.10-1.03)	0.07
**2021**				
Lowest	9/121	7.44 (2.81, 12.60)	Reference	
Medium	12/121	9.90 (4.59, 15.78)	1.01 (0.42-2.49)	0.99
Highest	10/124	8.06 (3.27 12.75)	0.92 (0.37-2.35)	0.87

Poisson regression models for the pooled analysis over all five surveys were adjusted for study year and the age of the child. Abbreviations: CI, confidence interval; EU, enzyme-linked immunosorbent assay unit.

Supplementary Table 6: Incidence of clinical malaria among children in the two intervention groups who received RTS,S/AS01$_E$ vaccine according to post-vaccination anti-CSP antibody cut-off titer defined by reverse cumulative plots, both countries combined (Burkina Faso and Mali), using the GSK ELISA.

Anti-CSP IgG antibody titer	Person years at risk (PYAR)	Events	Rate per 1000 PYAR (95% CI)	Hazard Ratio (95% CI)	P-value for threshold value
**Post-priming vaccination**					
Below threshold (266.8 EU/ml)	45.2	11	243.5 (121.6 to 435.7)	Reference	
Above threshold	123.7	5	40.4 (13.1 to 94.4)	0.13 (0.05 to 0.39)	<0.001
**Post-first booster dose**					
Below threshold (207.2 EU/ml)	99.1	30	302.7 (204.2 to 432.1)	Reference	
Above threshold	170.9	41	240.0 (172.2 to 325.5)	0.78 (0.45 to 1.36)	0.376
**Post-second booster dose**					
Below threshold (157.8 EU/ml)	113.9	41	359.8 (258.2 to 488.1)	Reference	
Above threshold	165.9	25	150.7 (97.5 to 222.5)	0.38 (0.21 to 0.69)	0.001
**Post-third booster dose**					
Below threshold (148.2 EU/ml)	166.8	47	281.8 (207.1 to 374.7)	Reference	
Above threshold	150.2	46	306.2 (224.2 to 408.5)	1.10 (0.68 to 1.80)	0.692
**Post-fourth booster dose**					
Below threshold (139.8 EU/ml)	186.9	83	444.1 (353.8 to 550.6)	Reference	
Above threshold	167.4	45	268.8 (196.1 to 359.7)	0.57 (0.37 to 0.90)	0.015
**Overall**					
Below threshold	611.9	212	346.5 (301.4 to 396.4)	Reference	
Above threshold	778	162	208.2 (177.4 to 242.9)	0.65 (0.51 to 0.83)	0.001

The Incidence of clinical malaria was compared between children with titers above and below a putative threshold protective titer, estimated from the reverse cumulative plots: 266.8 EU/ml in 2017, 207.2EU/ml in 2018, 157.8EU/ml in 2019, 148.2EU/ml in 2020 and 139.8EU/ml in 2021. The following efficacy of RTS,S /ASO1$_E$ against clinical episodes of malaria were used: 71.7% (95%CI 63.8 to 77.8) in the year after the three priming (2017) and 63.2% (95%CI 56.8 to 68.6), 58.6% (95%CI 51.5 to 64.6), 47.5% (95%CI 38.8 to 54.9) and 46.8% (95%CI 33.2 57.7) in the second (2018), third (2019), fourth (2020), and fifth year (2021) following subsequent boosting, respectively.

The overall analysis aggregates person-times and events above and below the specific threshold in each year of the study. The Cox regression models for the pooled analysis over all the five years of the study were adjusted for study year, country, and age of the child.

Supplementary Table 7: Logarithm of anti-CSP IgG antibody titres pre- and post-vaccination in each year in children who received RTS,S/AS01$_E$ vaccine, stratified by country assayed using the Oxford MSD assay.

Time of sample collection	N	Log GMT, EU/ml (95% CI)	Number with 2-fold increase in titre (%)	Number with 10-fold Increase in titre (%)
**Burkina Faso**				
Pre-2017	56	5.1 (5.1-5.1)		
Post-2017	65	12.2 (11.8-12.5)	61/65 (93.8)	54/65 (83.1)
Pre-2018	50	9.8 (9.4-10.1)		
Post-2018	50	12.2 (11.9-12.5)	50/50 (100)	26/50 (52)
Pre-2019	50	9.7 (9.5-9.9)		
Post-2019	50	11.1 (10.9-11.3)	40/50 (80)	7/50 (14)
Pre-2020	50	9.5 (9.2-9.8)		
Post-2020	50	10.3 (10.1-10.6)	28/50 (56)	0/50 (0)
Pre-2021	50	10.4 (10-10.8)		
Post-2021	50	10.7 (10.5-10.8)	17/50 (34)	1/50 (2)
**Mali**				
Pre-2017	48	5.3 (5.1-5.6)		
Post-2017	49	12 (11.6-12.4)	48/49 (98)	47/49 (95.9)
Pre-2018	49	8.5 (8-9.1)		
Post-2018	50	11.3 (11-11.6)	47/50 (94)	25/50 (50)
Pre-2019	50	9.9 (9.5-10.3)		
Post-2019	50	11.3 (11-11.6)	37/50 (74)	9/50 (18)
Pre-2020	49	9 (8.7-9.3)		
Post-2020	49	10.2 (10-10.5)	36/49 (73.5)	4/49 (8.2)
Pre-2021	50	9 (8.7-9.3)		
Post-2021	50	10.1 (9.8-10.4)	33/50 (66)	3/50 (6)

Supplementary Table 8: Logarithm of anti-CSP IgG antibody titers pre- and post-vaccination in each year in children who received the RTS,S/AS01$_E$ vaccine, stratified by country assayed using the GSK ELISA.

Time of sample collection	N	Log GMT, EU/ml (95% CI)	Number with 2-fold Increase in titer (%)	Number with 10-fold Increase in titer (%)
**Burkina Faso**				
Pre-2017[1]	201	-		
Post-2017	198	5.9 (5.8-6.1)	194/197 (98.5)	194/197 (98.5)
Pre-2018	279	3.6 (3.5-3.8)		
Post-2018	279	5.5 (5.4-5.6)	247/279 (88.5)	76/279 (27.2)
Pre-2019	291	3.8 (3.7-3.9)		
Post-2019	291	5.1 (5-5.2)	246/291 (84.5)	31/291 (10.7)
Pre-2020	327	3.6 (3.5-3.7)		
Post-2020	327	4.9 (4.8-4.9)	265/327 (81)	25/327 (7.6)
Pre-2021	385	3.6 (3.5-3.7)		
Post-2021	381	4.8 (4.7-4.9)	299/381 (78.5)	16/381 (4.2)
**Mali**				
Pre-2017	201	-		
Post-2017	198	5.9 (5.8-6.1)	194/197 (98.5)	194/197 (98.5)
Pre-2018	279	3.6 (3.5-3.8)		
Post-2018	279	5.5 (5.4-5.6)	247/279 (88.5)	76/279 (27.2)
Pre-2019	291	3.8 (3.7-3.9)		
Post-2019	291	5.1 (5-5.2)	246/291 (84.5)	31/291 (10.7)
Pre-2020	327	3.6 (3.5-3.7)		
Post-2020	327	4.9 (4.8-4.9)	265/327 (81)	25/327 (7.6)
Pre-2021	385	3.6 (3.5-3.7)		
Post-2021	381	4.8 (4.7-4.9)	299/381 (78.5)	16/381 (4.2)

[1] The lower limit of quantification for GSK ELISA is 1.9 EU/ml and samples with a titre below this lower limit (i.e. titre with a value of 0) were assigned a titre of 0.95 EU/ml, half the lower limit of detection. The log(0.95) = -0.051, many children have this value pre priming (in 2017) and it was not possible to calculate log GMT for these negative values.

Supplementary Table 9: Correlation coefficient between GSK ELISA and Oxford MSD ELISA titers pre- and post-vaccination, by country and calendar year.

**Variable**	**Pre-post titers combined**		**Pre-titer**		**Post-titer**	
	**No.**	**r (95% CI)**	**No.**	**r (95% CI)**	**No.**	**r (95% CI)**
Both Countries	1015	0.94 (0.93-0.95)	502	0.93 (0.92-0.94)	513	0.85 (0.82-0.87)
Country						
Burkina Faso	521	0.95 (0.95-0.96)	256	0.96 (0.95-0.97)	265	0.83 (0.79-0.87)
Mali	494	0.93 (0.92-0.94)	246	0.89 (0.87-0.92)	248	0.87 (0.83-0.89)
Year						
2017	218	0.98 (0.97-0.98)	104	-	114	0.78 (0.69-0.84)
2018	199	0.85 (0.80-0.88)	99	0.70 (0.58-0.79)	100	0.74 (0.64-0.82)
2019	200	0.93 (0.91-0.95)	100	0.94 (0.91-0.96)	100	0.84 (0.77-0.89)
2020	198	0.94 (0.92-0.95)	99	0.93 (0.90-0.95)	99	0.91 (0.86-0.94)
2021	200	0.78 (0.72-0.83)	100	0.78 (0.69-0.85)	100	0.84 (0.77-0.89)

r, rho, Pearson correlation coefficient on the log10 transformed titers.

Supplementary Table 10. Overview of study design and groups with dates of administration of vaccine.

<u>Year</u>	**Group A**<br>SMC alone	**Group B**<br>RTS,S/AS01$_E$ alone	**Group C**<br>RTS,S/AS01$_E$ + SMC
	**Year 1 – 2017 (Primary series)**		
April – June – July	Rabies x3	RTS,S/AS01$_E$ x3	RTS,S/AS01$_E$ x3
July – August – September - October	SMC* x4	SMC* Placebo x4	SMC* x4
	**Year 2 – 2018 (Booster dose1)**		
June	Rabies x1	RTS,S/AS01$_E$ x1	RTS,S/AS01$_E$ x1
July – August – September - October	SMC* x4	SMC* Placebo x4	SMC* x4
	**Year 3 – 2019 (Booster dose 2)**		
June	Rabies x1	RTS,S/AS01$_E$ x1	RTS,S/AS01$_E$ x1
July – August – September - October	SMC* x4	SMC* Placebo x4	SMC* x4
	**Year 4 – 2019 (Booster dose 3)**		
June	Rabies x1	RTS,S/AS01$_E$ x1	RTS,S/AS01$_E$ x1
July – August – September - October	SMC* x4	SMC* Placebo x4	SMC* x4
	**Year 4 – 2019 (Booster dose 4)**		
June	Rabies x1	RTS,S/AS01$_E$ x1	RTS,S/AS01$_E$ x1
July – August – September - October	SMC* x4	SMC* Placebo x4	SMC* x4

Supplementary Figure 1: Anti-CSP antibody titres in individual children pre- and post-priming vaccination (2017), pre and post first (2018), second (2019), third (2020), and fourth (0221) booster seasonal vaccination doses shown by country. Results from children in the RTS,S/AS01$_E$ alone group are shown in blue, those from children in the combined group are shown in red. Abbreviations: CSP, circumsporozoite; EU, enzyme-linked immunosorbent assay unit.

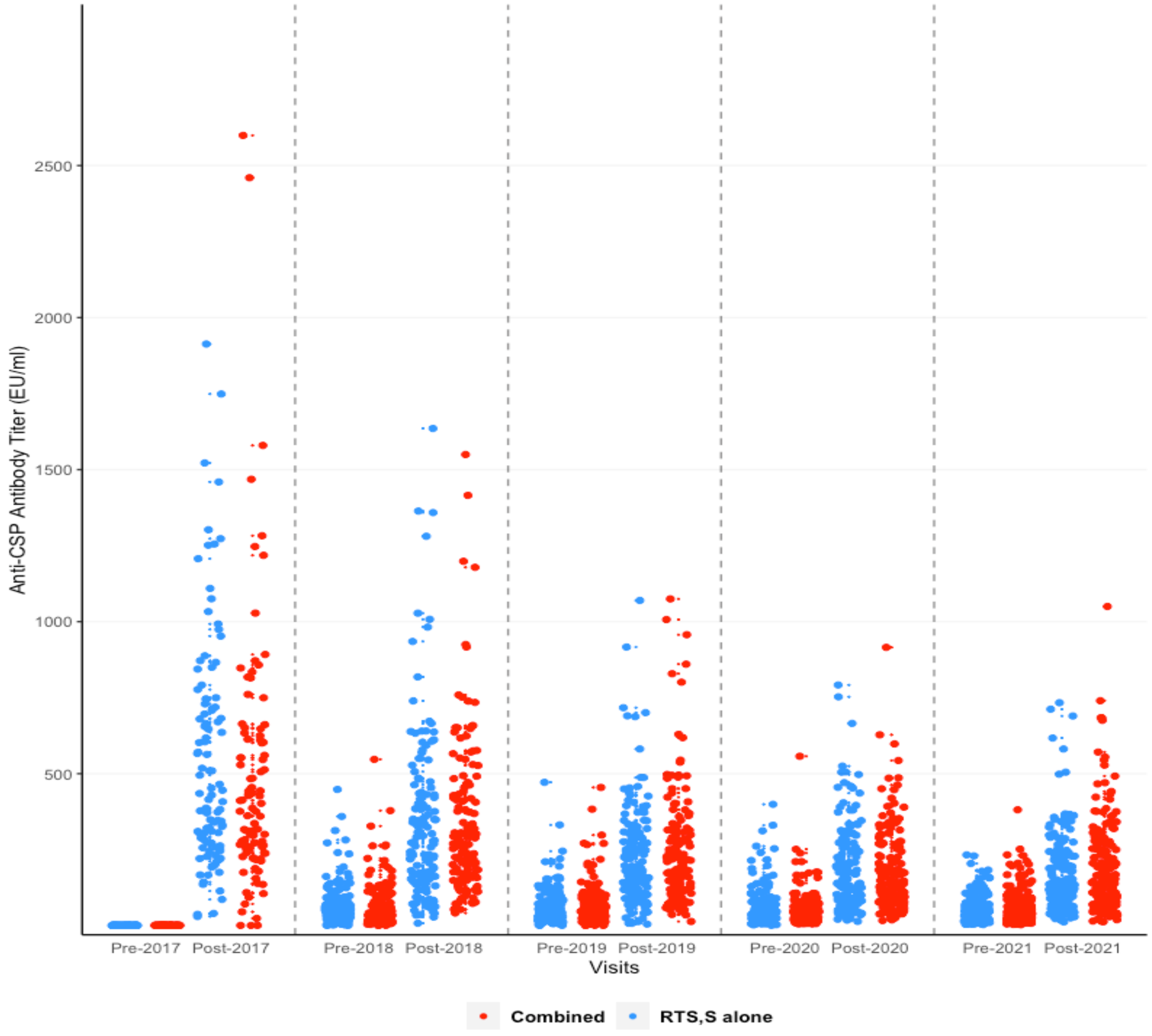

Supplementary Figure 2: Reverse cumulative plots of antibody titre by study year, country, and study arm using GSK ELISA. The first five set of panels shows titres by study group RTSS/AS01E alone group (red line) combined intervention group (blue line). The second five set of panels shows titres by study country—Burkina Faso (blue line), Mali (red line). Abbreviations: CSP, circumsporozoite; EU, enzyme-linked immunosorbent assay unit.

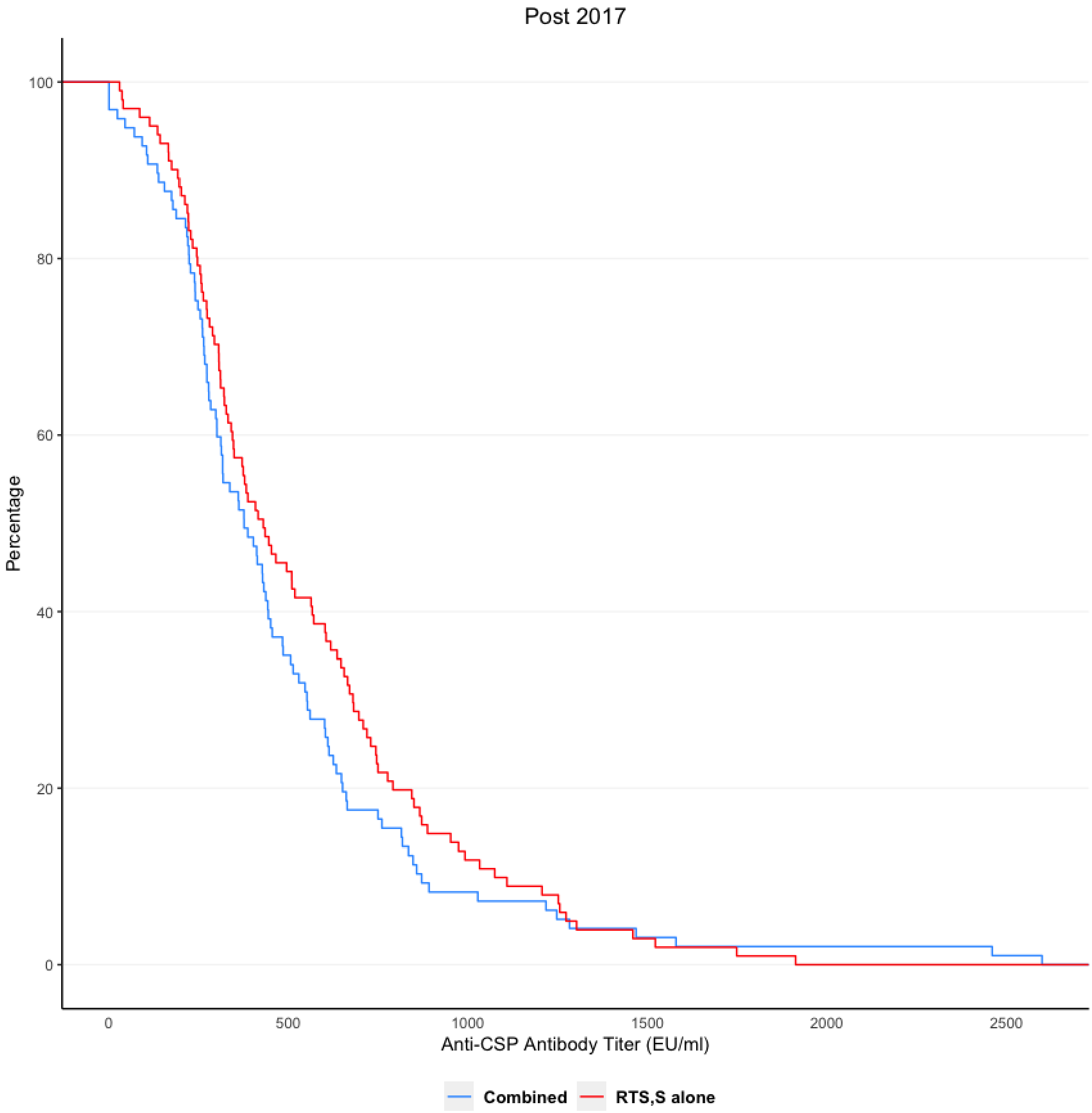

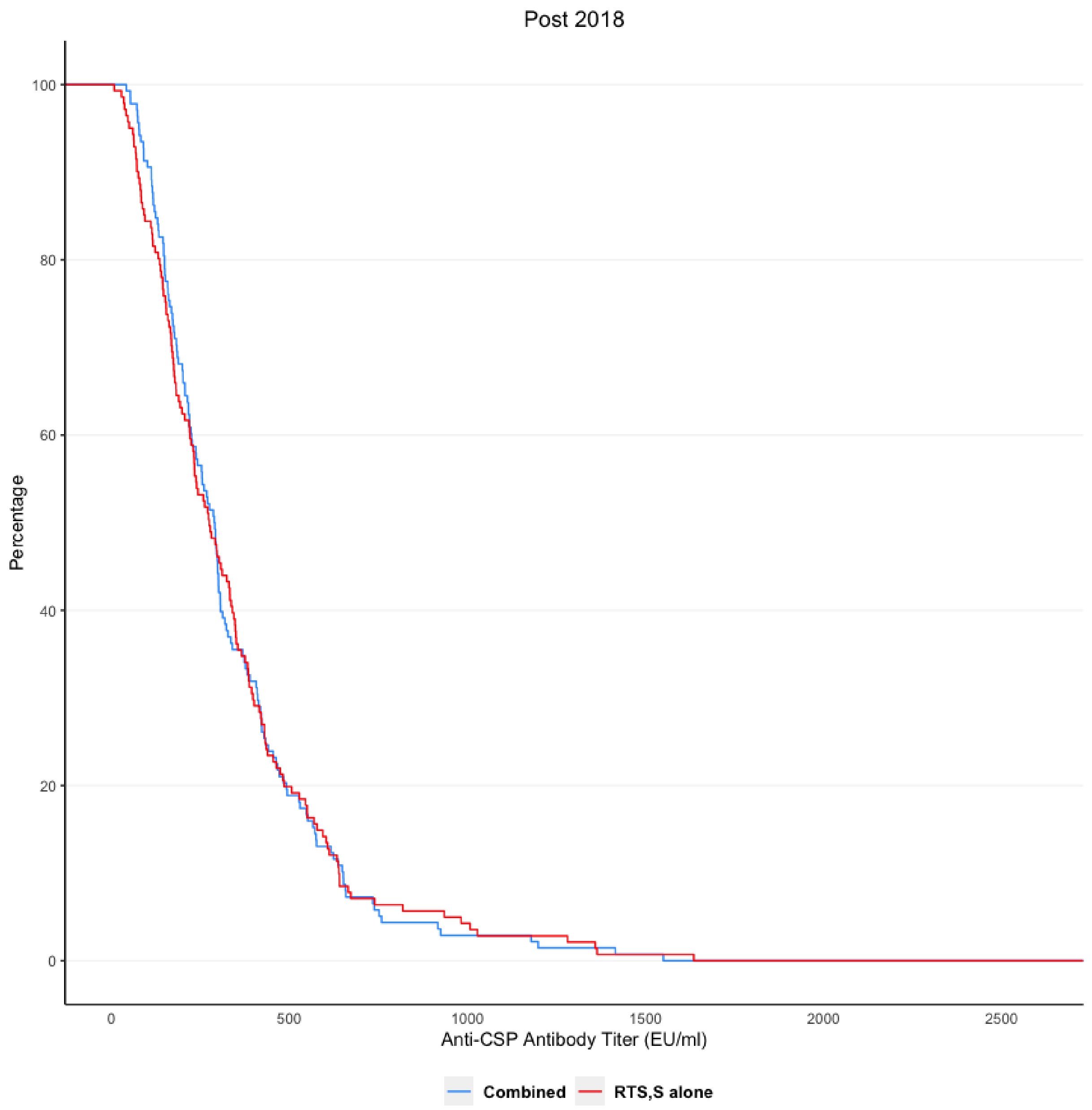

Post 2018
Percentage
100
80
60
40
20
0
0
500
1000
1500
2000
2500
Anti-CSP Antibody Titer (EU/ml)
Combined
RTS,S alone

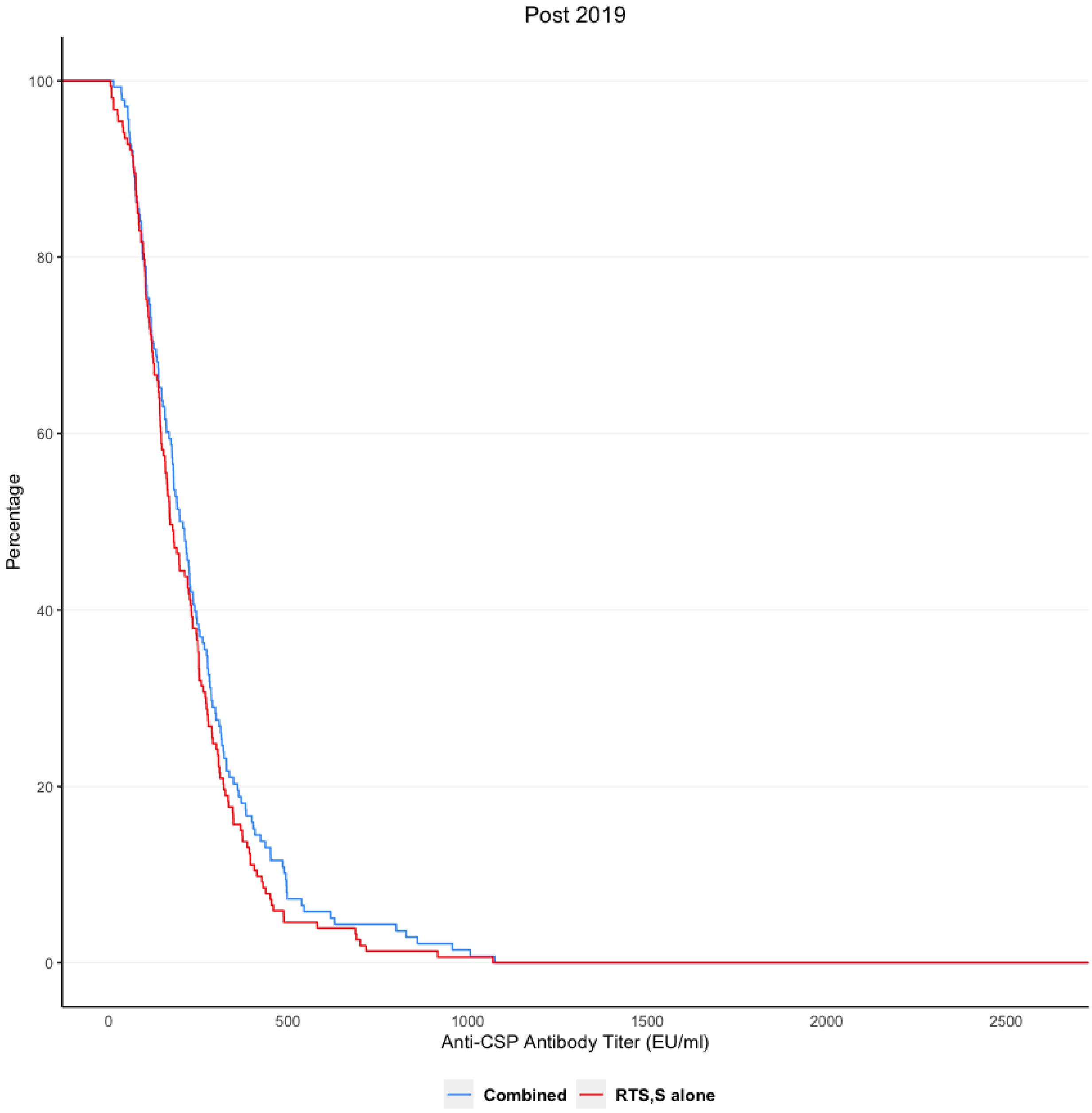
Post 2019
Percentage
100
80
60
40
20
0
0
500
1000
1500
2000
2500
Anti-CSP Antibody Titer (EU/ml)
Combined
RTS,S alone

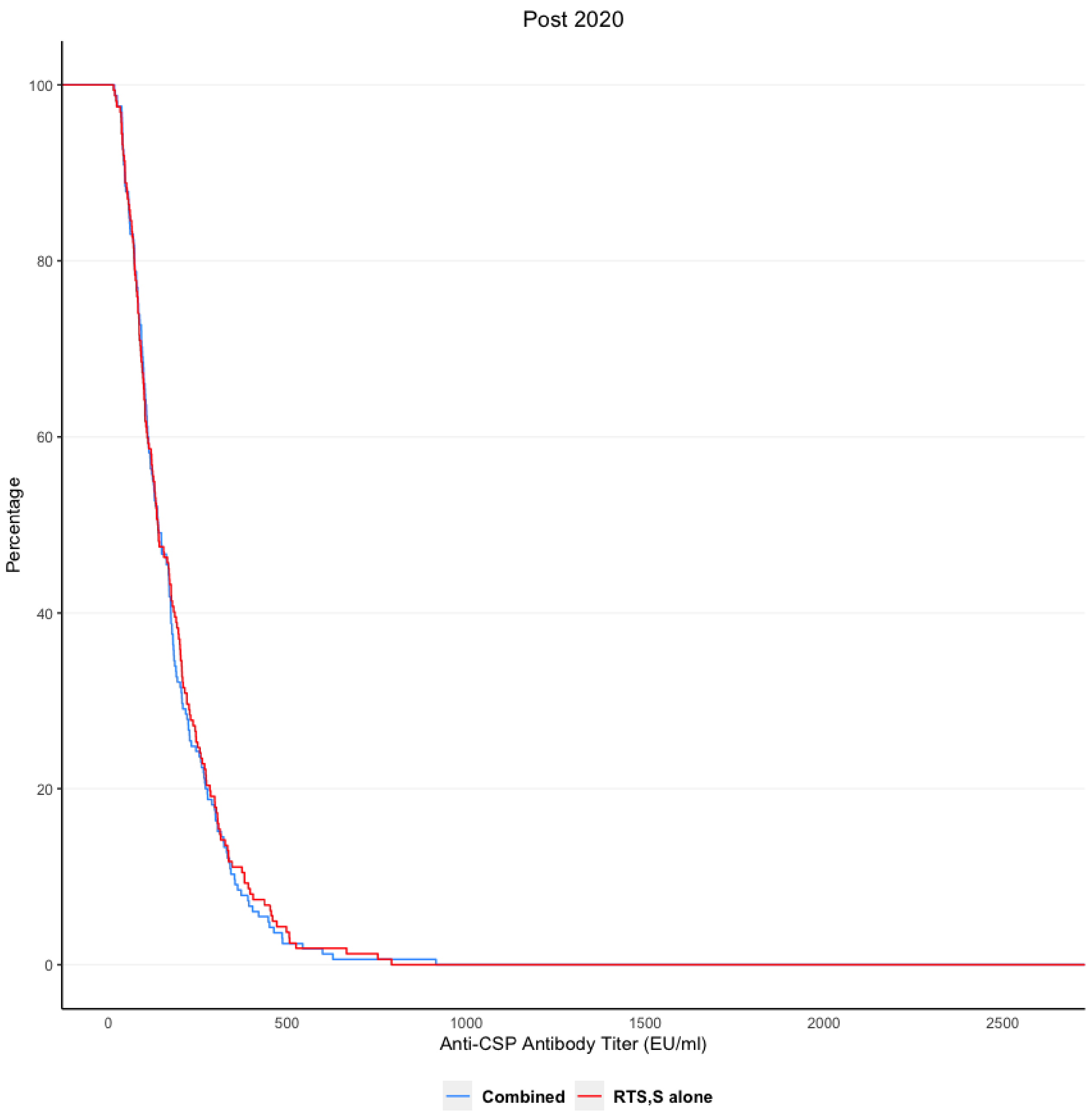

Post 2020
Percentage
100
80
60
40
20
0
0
500
1000
1500
2000
2500
Anti-CSP Antibody Titer (EU/ml)
Combined
RTS,S alone

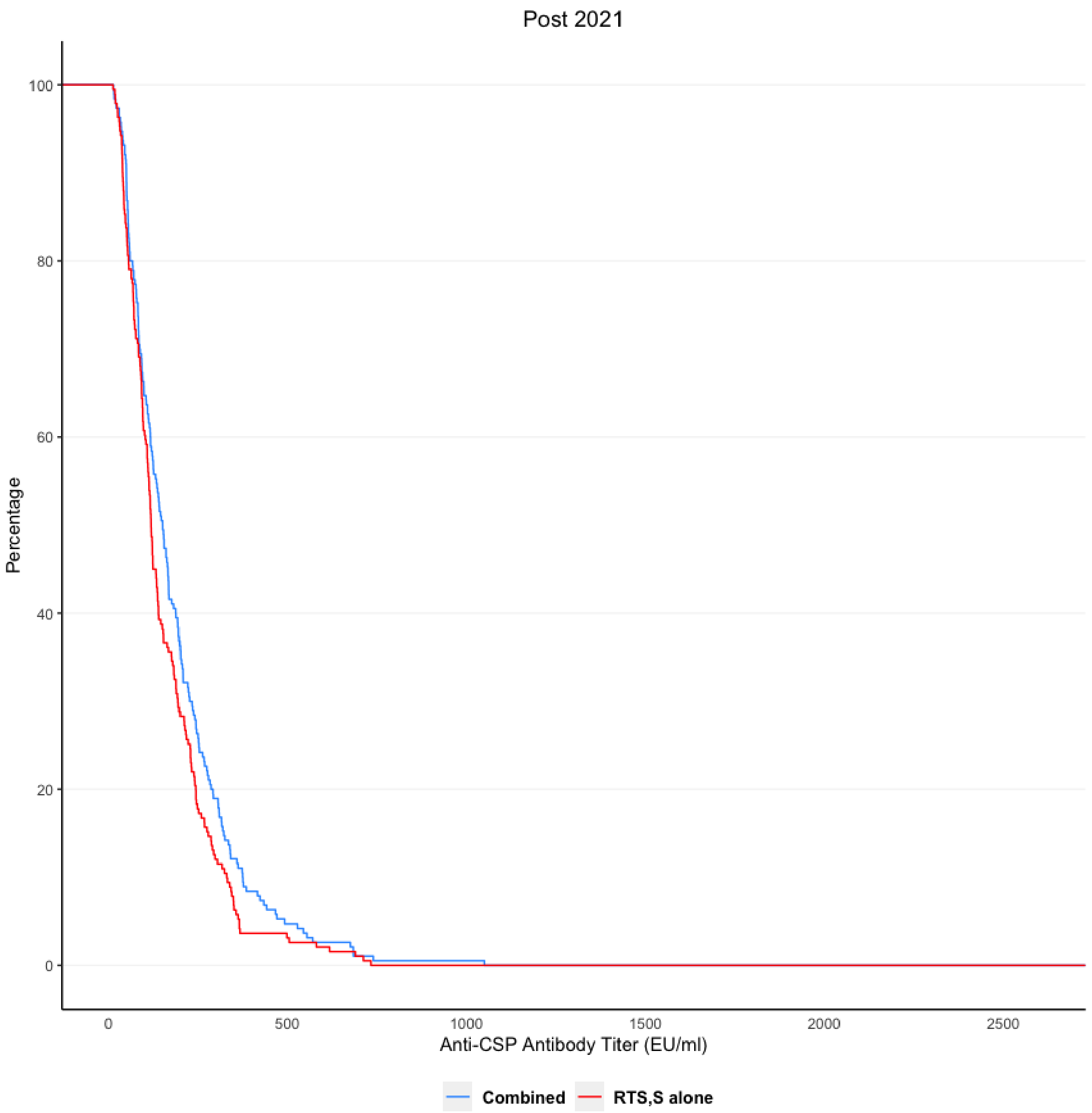

Post 2021
Percentage
100
80
60
40
20
0
0
500
1000
1500
2000
2500
Anti-CSP Antibody Titer (EU/ml)
Combined
RTS,S alone

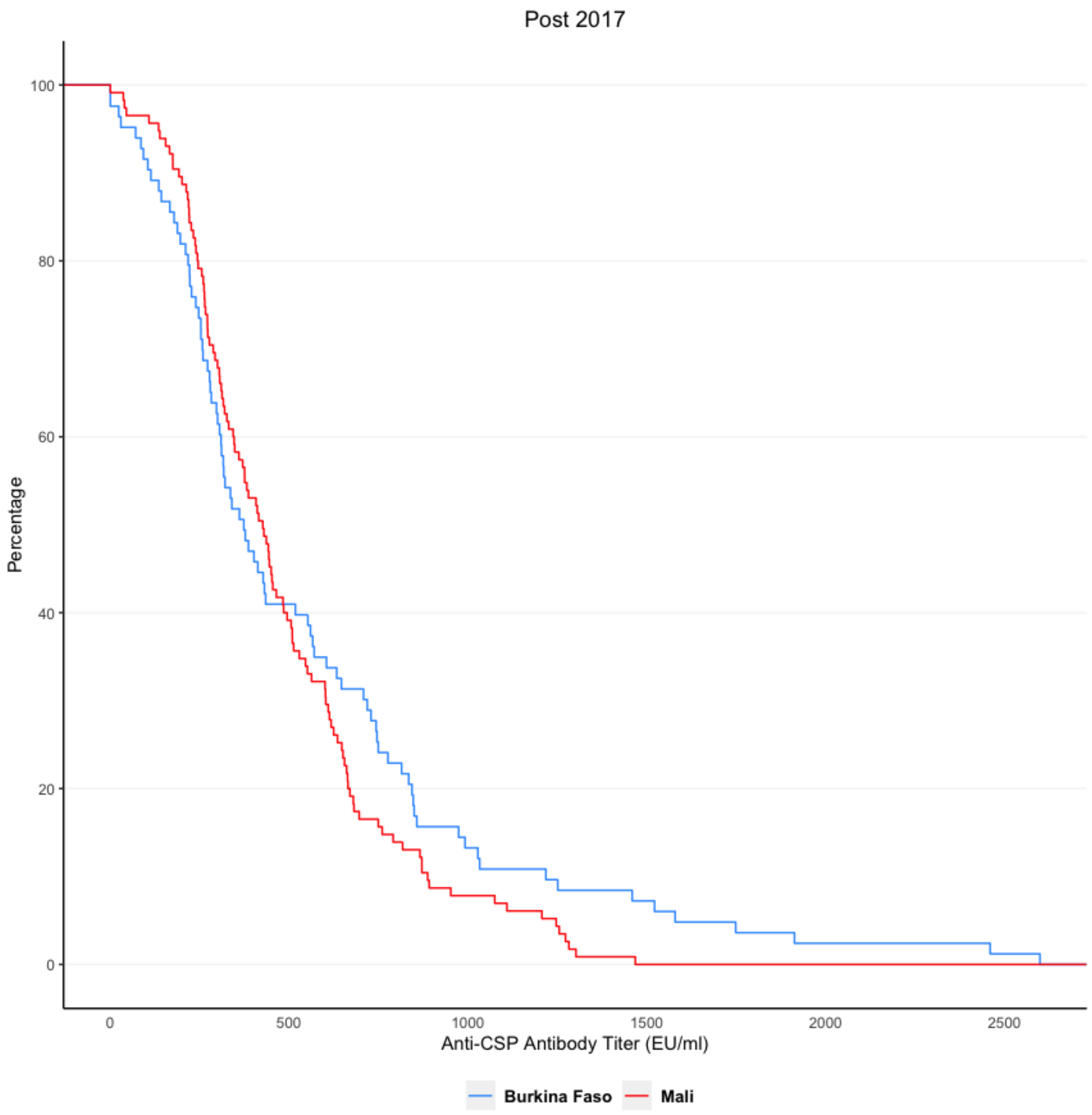

Post 2017
Percentage
100
80
60
40
20
0
0
500
1000
1500
2000
2500
Anti-CSP Antibody Titer (EU/ml)
Burkina Faso
Mali

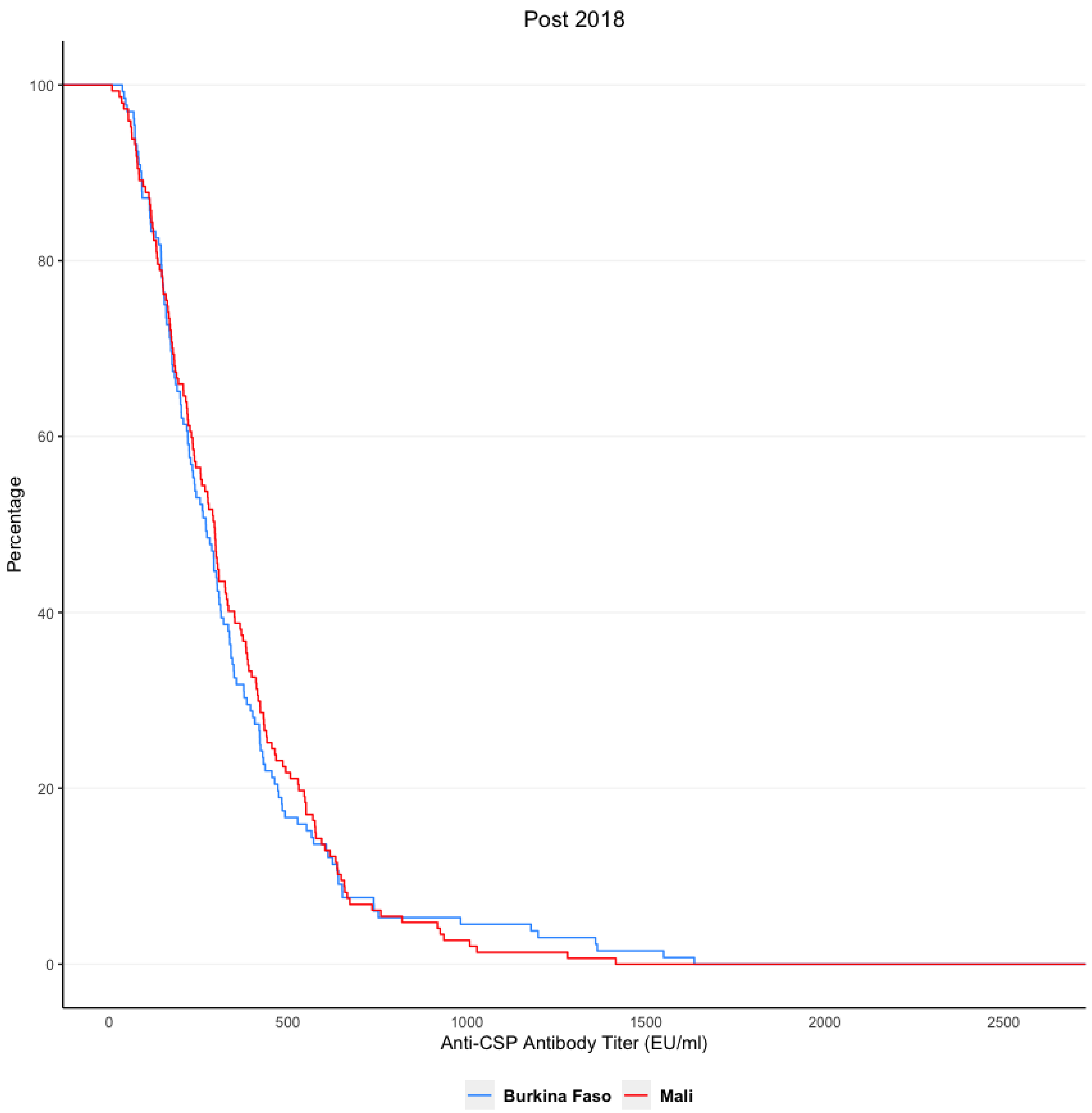

Post 2018
Percentage
100
80
60
40
20
0
0
500
1000
1500
2000
2500
Anti-CSP Antibody Titer (EU/ml)
Burkina Faso
Mali

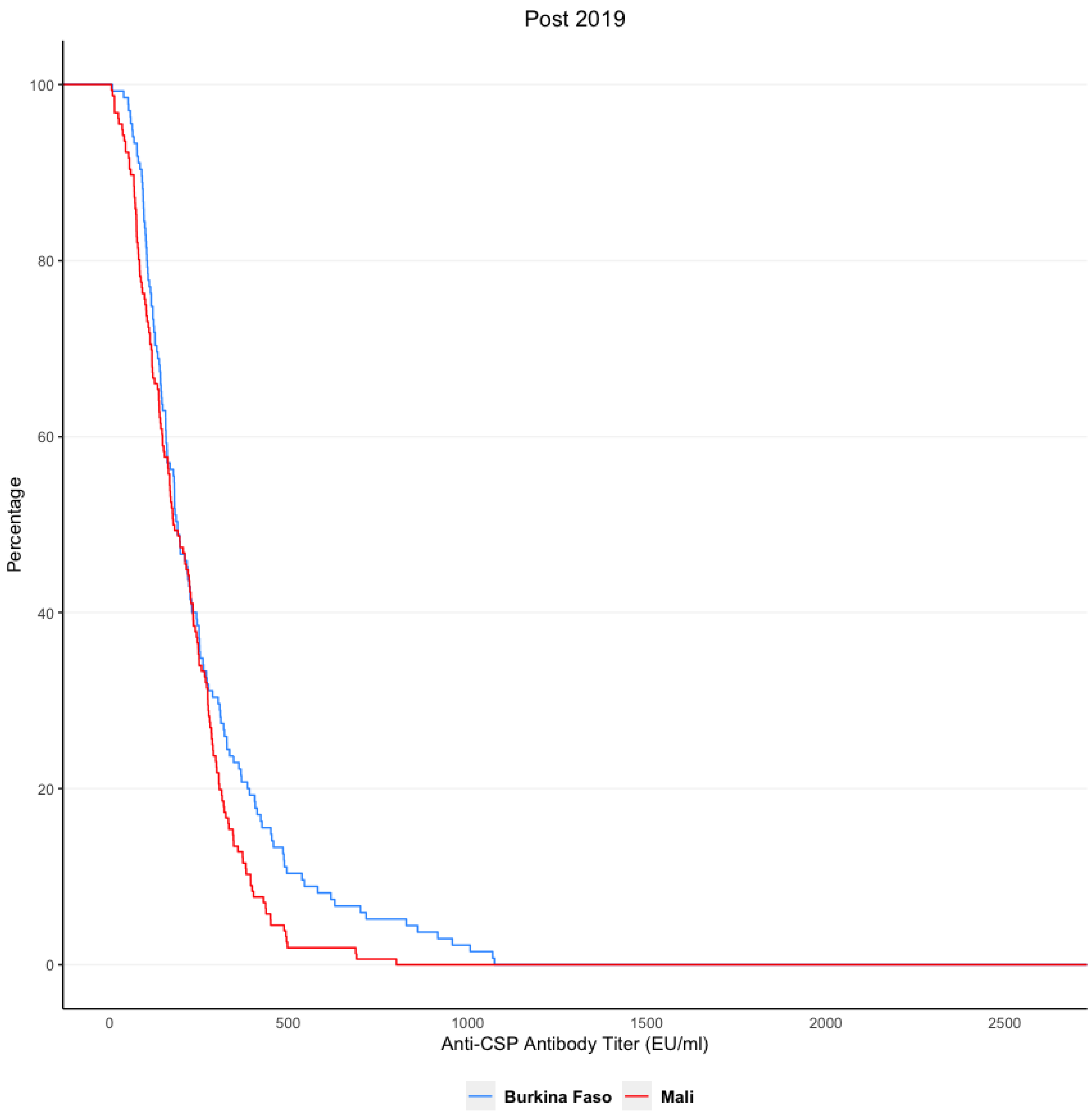

Post 2019
Percentage
100
80
60
40
20
0
0
500
1000
1500
2000
2500
Anti-CSP Antibody Titer (EU/ml)
Burkina Faso
Mali

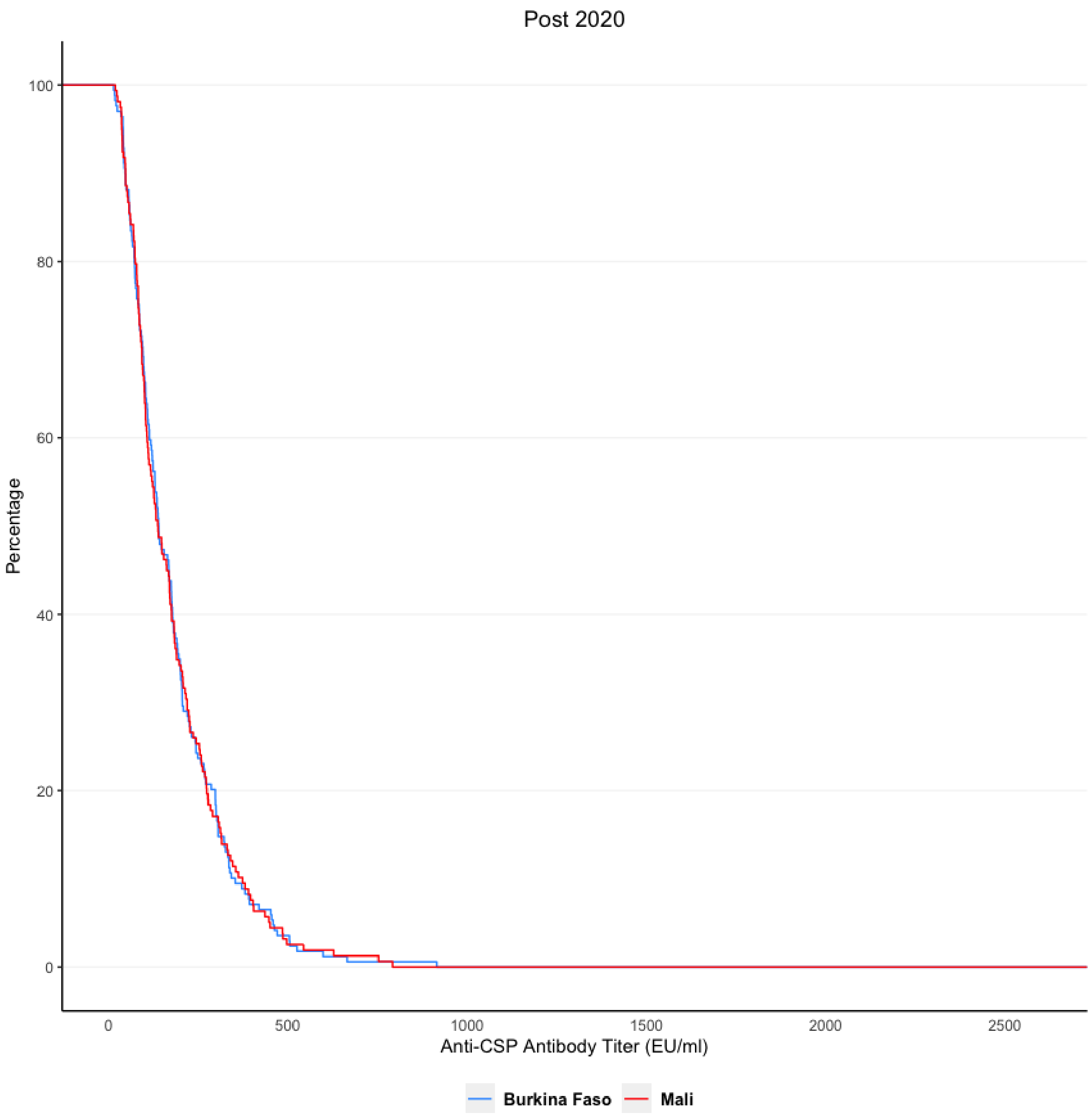
Post 2020
Percentage
100
80
60
40
20
0
0
500
1000
1500
2000
2500
Anti-CSP Antibody Titer (EU/ml)
Burkina Faso
Mali

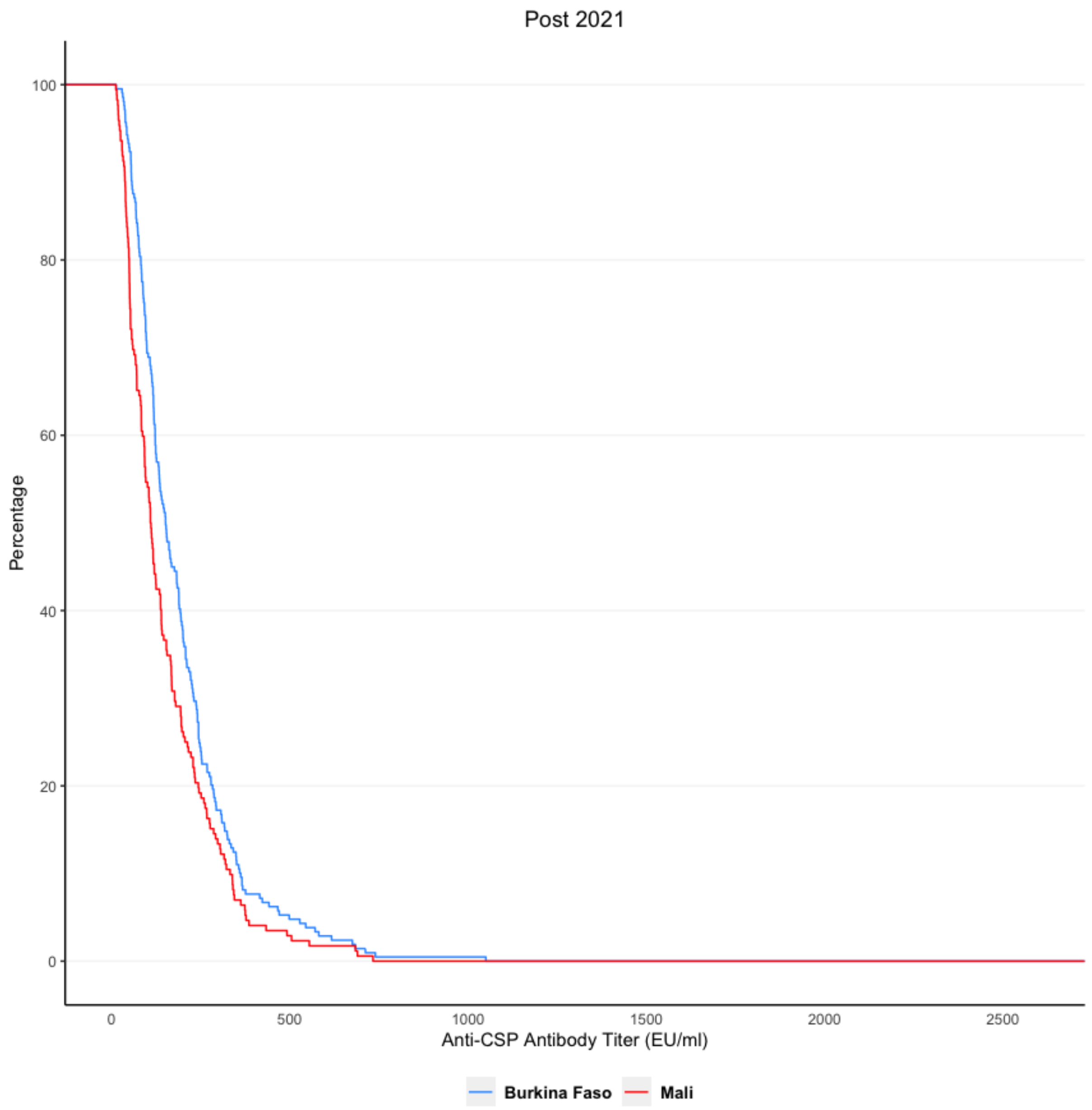
Post 2021
Percentage
100
80
60
40
20
0
0
500
1000
1500
2000
2500
Anti-CSP Antibody Titer (EU/ml)
Burkina Faso
Mali

Check list developed for the submission to the Lancet Infectious Diseases to accompany the paper describing the main results for the RTS,S, +SMC trial during which the samples described in the immunology sub-study were obtained. The reference for this paper is Dicko, A. et al. Seasonal vaccination with RTS, S/AS01E vaccine with or without seasonal malaria chemoprevention in children up to the age of 5 years in Burkina Faso and Mali: a double-blind, randomised, controlled, phase 3 trial. *Lancet Infect. Dis*. **24**, 75-86 (2024).

Reporting checklist for randomised trial.

Based on the CONSORT guidelines.

Instructions to authors

Complete this checklist by entering the page numbers from your manuscript where readers will find each of the items listed below.

Your article may not currently address all the items on the checklist. Please modify your text to include the missing information. If you are certain that an item does not apply, please write "n/a" and provide a short explanation.

Upload your completed checklist as an extra file when you submit to a journal.

In your methods section, say that you used the CONSORTreporting guidelines, and cite them as:

Schulz KF, Altman DG, Moher D, for the CONSORT Group. CONSORT 2010 Statement: updated guidelines for reporting parallel group randomised trials

		Reporting Item	Page Number
**Title and Abstract**			
Title	#1a	Identification as a randomized trial in the title.	1
Abstract	#1b	Structured summary of trial design, methods, results, and conclusions	2
**Introduction**			

Background and objectives	#2a	Scientific background and explanation of rationale	4
Background and objectives	#2b	Specific objectives or hypothesis	4
**Methods**			
Trial design	#3a	Description of trial design (such as parallel, factorial) including allocation ratio.	4
Trial design	#3b	Important changes to methods after trial commencement (such as eligibility criteria), with reasons	
Participants	#4a	Eligibility criteria for participants	5
Participants	#4b	Settings and locations where the data were collected	4
Interventions	#5	The experimental and control interventions for each group with sufficient details to allow replication, including how and when they were actually administered	5
Outcomes	#6a	Completely defined prespecified primary and secondary outcome measures, including how and when they were assessed	5-6
Outcomes	#6b	Any changes to trial outcomes after the trial commenced, with reasons	6
Sample size	#7a	How sample size was determined.	6
Sample size	#7b	When applicable, explanation of any interim analyses and stopping guidelines	
Randomization - Sequence generation	#8a	Method used to generate the random allocation sequence.	5

Randomization - Sequence generation	#8b	Type of randomization; details of any restriction (such as blocking and block size)	5
Randomization - Allocation concealment mechanism	#9	Mechanism used to implement the random allocation sequence (such as sequentially numbered containers), describing any steps taken to conceal the sequence until interventions were assigned	5
Randomization - Implementation	#10	Who generated the allocation sequence, who enrolled participants, and who assigned participants to interventions	5
Blinding	#11a	If done, who was blinded after assignment to interventions (for example, participants, care providers, those assessing outcomes) and how.	5
Blinding	#11b	If relevant, description of the similarity of interventions	5
Statistical methods	#12a	Statistical methods used to compare groups for primary and secondary outcomes	6,7
Statistical methods	#12b	Methods for additional analyses, such as subgroup analyses and adjusted analyses	6,7
**Results**			
Participant flow diagram (strongly recommended)	#13a	For each group, the numbers of participants who were randomly assigned, received intended treatment, and were analysed for the primary outcome	7, Fig 2
Participant flow	#13b	For each group, losses and exclusions after randomization, together with reason	7, Fig 2
Recruitment	#14a	Dates defining the periods of recruitment and follow-up	5, Fig 1

Recruitment	#14b	Why the trial ended or was stopped	7
Baseline data	#15	A table showing baseline demographic and clinical characteristics for each group	data are published in NEJM manuscript that reported the results from the first three years of the study.p13 ref 6.
Numbers analysed	#16	For each group, number of participants (denominator) included in each analysis and whether the analysis was by original assigned groups	7-9
Outcomes and estimation	#17a	For each primary and secondary outcome, results for each group, and the estimated effect size and its precision (such as 95% confidence interval)	7-9
Outcomes and estimation	#17b	For binary outcomes, presentation of both absolute and relative effect sizes is recommended	8-9
Ancillary analyses	#18	Results of any other analyses performed, including subgroup analyses and adjusted analyses, distinguishing pre-specified from exploratory	8-9
Harms	#19	All important harms or unintended effects in each group (For specific guidance see CONSORT for harms)	9
**Discussion**			
Limitations	#20	Trial limitations, addressing sources of potential bias, imprecision, and, if relevant, multiplicity of analyses	10
Generalisability	#21	Generalisability (external validity, applicability) of the trial findings	9 - 11
Interpretation	#22	Interpretation consistent with results, balancing benefits and harms, and considering other relevant evidence	9 - 11

Registration	#23	Registration number and name of trial registry	2, 7
**Other information**			
Interpretation	#22	Interpretation consistent with results, balancing benefits and harms, and considering other relevant evidence	9 - 10
Registration	#23	Registration number and name of trial registry	2, 7
Protocol	#24	Where the full trial protocol can be accessed, if available	4
Funding	#25	Sources of funding and other support (such as supply of drugs), role of funders	7, 12

None The CONSORT checklist is distributed under the terms of the Creative Commons Attribution License CC-BY. This checklist can be completed online using https://www.goodreports.org/, a tool made by the EQUATOR Network in collaboration with Penelope.ai

Check list for the paper submitted to npj vaccines describing the immunology sub-study of the RTS,S +SMC trial described in Dicko, A. et al. Seasonal vaccination with RTS, S/AS01E vaccine with or without seasonal malaria chemoprevention in children up to the age of 5 years in Burkina Faso and Mali: a double-blind, randomised, controlled, phase 3 trial. *Lancet Infect. Dis*. **24**, 75-86 (2024).

Reporting checklist for randomised trial.

Based on the CONSORT guidelines.

Instructions to authors

Complete this checklist by entering the page numbers from your manuscript where readers will find each of the items listed below.

Your article may not currently address all the items on the checklist. Please modify your text to include the missing information. If you are certain that an item does not apply, please write "n/a" and provide a short explanation.

Upload your completed checklist as an extra file when you submit to a journal.

In your methods section, say that you used the CONSORTreporting guidelines, and cite them as:

Schulz KF, Altman DG, Moher D, for the CONSORT Group. CONSORT 2010 Statement: updated guidelines for reporting parallel group randomised trials

		Reporting Item	Page Number
**Title and Abstract**			
Title	#1a	Identification as a randomized trial in the title.	N/A
Abstract	#1b	Structured summary of trial design, methods, results, and conclusions	Page 2 (No headings in line with journal format)
**Introduction**			
Background and objectives	#2a	Scientific background and explanation of rationale	Pages 2 and 3
Background and objectives	#2b	Specific objectives or hypothesis	Page 3

Methods

Trial design	#3a	Description of trial design (such as parallel, factorial) including allocation ratio.	Covered for the main trial in refs 5 and 6
Trial design	#3b	Important changes to methods after trial commencement (such as eligibility criteria), with reasons	N/A
Participants	#4a	Eligibility criteria for participants	Page 10
Participants	#4b	Settings and locations where the data were collected	Page 10
Interventions	#5	The experimental and control interventions for each group with sufficient details to allow replication, including how and when they were actually administered	Page 10
Outcomes	#6a	Completely defined prespecified primary and secondary outcome measures, including how and when they were assessed	Covered for the main trial in refs 5 and 6
Outcomes	#6b	Any changes to trial outcomes after the trial commenced, with reasons	N/A
Sample size	#7a	How sample size was determined.	Page 12
Sample size	#7b	When applicable, explanation of any interim analyses and stopping guidelines	N/A
Randomization - Sequence generation	#8a	Method used to generate the random allocation sequence.	Page 9
Randomization - Sequence generation	#8b	Type of randomization; details of any restriction (such as blocking and block size)	Page 9
Randomization - Allocation concealment mechanism	#9	Mechanism used to implement the random allocation sequence (such as sequentially numbered containers), describing any steps taken to conceal the sequence until interventions were assigned	Covered for the main trial in refs 5 and 6

Randomization - Implementation	#10	Who generated the allocation sequence, who enrolled participants, and who assigned participants to interventions	Page 10
Blinding	#11a	If done, who was blinded after assignment to interventions (for example, participants, care providers, those assessing outcomes) and how.	Page 12
Blinding	#11b	If relevant, description of the similarity of interventions	N/A
Statistical methods	#12a	Statistical methods used to compare groups for primary and secondary outcomes	Pages 12 and 13
Statistical methods	#12b	Methods for additional analyses, such as subgroup analyses and adjusted analyses	N/A
**Results**			
Participant flow diagram (strongly recommended)	#13a	For each group, the numbers of participants who were randomly assigned, received intended treatment, and were analysed for the primary outcome	Page 18<br>Table 1
Participant flow	#13b	For each group, losses and exclusions after randomization, together with reason	Covered for the main trial in refs 5 and 6
Recruitment	#14a	Dates defining the periods of recruitment and follow-up	Covered for the main trial in refs 5 and 6
Recruitment	#14b	Why the trial ended or was stopped	Covered for the main trial in refs 5 and 6
Baseline data	#15	A table showing baseline demographic and clinical characteristics for each group	Page 18<br>Table 1
Numbers analysed	#16	For each group, number of participants (denominator) included in each analysis and whether the analysis was by original assigned groups	N/A

Outcomes and estimation	#17a	For each primary and secondary outcome, results for each group, and the estimated effect size and its precision (such as 95% confidence interval)	Covered for the main trial in refs 5 and 6
Outcomes and estimation	#17b	For binary outcomes, presentation of both absolute and relative effect sizes is recommended	Covered for the main trial in refs 5 and 6
Ancillary analyses	#18	Results of any other analyses performed, including subgroup analyses and adjusted analyses, distinguishing pre-specified from exploratory	N/A
Harms	#19	All important harms or unintended effects in each group (For specific guidance see CONSORT for harms)	Covered for the main trial in refs 5 and 6
**Discussion**			
Limitations	#20	Trial limitations, addressing sources of potential bias, imprecision, and, if relevant, multiplicity of analyses	Page 8
Generalisability	#21	Generalisability (external validity, applicability) of the trial findings	N/A
Interpretation	#22	Interpretation consistent with results, balancing benefits and harms, and considering other relevant evidence	N/A
Registration	#23	Registration number and name of trial registry	Page 13
**Other information**			
Interpretation	#22	Interpretation consistent with results, balancing benefits and harms, and considering other relevant evidence	N/A
Registration	#23	Registration number and name of trial registry	Page 13
Protocol	#24	Where the full trial protocol can be accessed, if available	Page 13
Funding	#25	Sources of funding and other support (such as supply of drugs), role of funders	Pages 14 and 15

None The CONSORT checklist is distributed under the terms of the Creative Commons Attribution License CC-BY. This checklist can be completed online using https://www.goodreports.org/, a tool made by the EQUATOR Network in collaboration with Penelope.ai
